# *Slc20a2*, Encoding the Phosphate Transporter PiT2, Is an Important Genetic Determinant of Bone Quality and Strength

**DOI:** 10.1002/jbmr.3691

**Published:** 2019-03-19

**Authors:** Sarah Beck‐Cormier, Christopher J Lelliott, John G Logan, David T Lafont, Laure Merametdjian, Victoria D Leitch, Natalie C Butterfield, Hayley J Protheroe, Peter I Croucher, Paul A Baldock, Alina Gaultier‐Lintia, Yves Maugars, Gael Nicolas, Christopher Banse, Sébastien Normant, Nicolas Magne, Emmanuel Gérardin, Nina Bon, Sophie Sourice, Jérôme Guicheux, Laurent Beck, Graham R Williams, J H Duncan Bassett

**Affiliations:** ^1^ INSERM, UMR 1229, Regenerative Medicine and Skeleton (RMeS), Université de Nantes, École Nationale Vétérinaire, Agroalimentaire et de l'Alimentation Nantes‐Atlantique (ONIRIS) Nantes France; ^2^ Université de Nantes Unité de Formation et de Recherche (UFR) Odontologie Nantes France; ^3^ Mouse Pipelines Wellcome Trust Sanger Institute Hinxton UK; ^4^ Molecular Endocrinology Laboratory Department of Medicine Imperial College London London UK; ^5^ Centre Hospitalier Universitaire (CHU) Nantes Pôles Hospitalo‐Universitaires (PHU4) ‐ Ostéo‐articulaire ‐ Tête et Cou ‐ Odontologie ‐ Neurochirurgie ‐ Neuro‐traumatologie (OTONN) Nantes France; ^6^ The Garvan Institute of Medical Research Sydney NSW Australia; ^7^ St Vincent's Clinical School, Faculty of Medicine University of New South Wales (UNSW) Australia Sydney NSW Australia; ^8^ Centre Hospitalier Universitaire (CHU) Nantes Laennec Hospital Nantes France; ^9^ INSERM U1245 Université de Rouen Normandie (UNIROUEN) Rouen France; ^10^ Department of Genetics Rouen University Hospital Rouen France; ^11^ Centre National de Référence pour les Malades Alzheimer Jeunes (CNR‐MAJ) Normandy Center for Genomic and Personalized Medicine Rouen France; ^12^ Department of Rheumatology Soissons Hospital Soissons France; ^13^ Department of Neuroradiology Rouen University Hospital Rouen France

**Keywords:** ANIMAL MODELS (GENETIC ANIMAL MODELS), BONE MATRIX (MATRIX MINERALIZATION), DISORDERS OF CALCIUM/PHOSPHATE METABOLISM (OTHER), GENETIC RESEARCH (HUMAN ASSOCIATION STUDIES), ORTHOPAEDICS (BIOMECHANICS)

## Abstract

Osteoporosis is characterized by low bone mineral density (BMD) and fragility fracture and affects over 200 million people worldwide. Bone quality describes the material properties that contribute to strength independently of BMD, and its quantitative analysis is a major priority in osteoporosis research. Tissue mineralization is a fundamental process requiring calcium and phosphate transporters. Here we identify impaired bone quality and strength in *Slc20a2^–/–^* mice lacking the phosphate transporter SLC20A2. Juveniles had abnormal endochondral and intramembranous ossification, decreased mineral accrual, and short stature. Adults exhibited only small reductions in bone mass and mineralization but a profound impairment of bone strength. Bone quality was severely impaired in *Slc20a2^–/–^* mice: yield load (–2.3 SD), maximum load (–1.7 SD), and stiffness (–2.7 SD) were all below values predicted from their bone mineral content as determined in a cohort of 320 wild‐type controls. These studies identify *Slc20a2* as a physiological regulator of tissue mineralization and highlight its critical role in the determination of bone quality and strength. © 2019 The Authors. *Journal of Bone and Mineral Research* Published by Wiley Periodicals Inc.

## Introduction

Osteoporosis is a common age‐related disorder characterized by low bone mineral density (BMD) and a loss of structural integrity leading to an increased susceptibility to fragility fracture. Osteoporosis affects over 200 million people worldwide with over 9 million fractures occurring annually that result in a massive and increasing healthcare burden.^1^ Although measurement of BMD by bone densitometry (DXA) is a good predictor of fracture risk, it fails to account for the effects of bone quality. Bone quality is the term used to describe properties of bone composition and structure that contribute to strength independently of BMD and it has emerged as an important priority in osteoporosis research.^2^ Thus, the material properties and strength of the skeleton reflect contributions from both bone quality and tissue mineralization.

Bone mineralization is a tightly regulated process involving progressive mineralization of collagen fibrils secreted by bone‐forming osteoblasts. Although the mechanisms of apatite crystal formation remain controversial, matrix vesicle (MV) budding from the plasma membrane of mineralizing cells (osteoblasts or mature chondrocytes) has been shown to create a nucleation space for apatite crystals that deposit on collagen fibrils after MV rupture.^3^, ^4^ This process requires that calcium (Ca) and phosphate (Pi) transporters are expressed within MVs, whereas obligatory expression of transporters at the cell surface is further supported by the formation of transient intracellular amorphous mineral precursors within mineralizing cells.^5^ In mammals, PiT1/SLC20A1 and PiT2/SLC20A2 are the major Pi transporters expressed along with NPT2A in skeletal cells, and they are thought to have a pivotal role in providing Pi for mineralization.^6^, ^7^ Initial in vitro observations indicated that SLC20A1 expression was regulated by osteogenic factors,^(8–13)^ suggesting a specific role for SLC20A1 in skeletal mineralization.^10^, ^14^ However, more recent in vivo approaches challenged this hypothesis as (i) *Slc20a1* hypomorphic adult mice had normal bone mineralization,^15^ (ii) chondrocyte‐specific deletion (using Col2a‐Cre or Agc1‐CreER^T2^ mice) of *Slc20a1* revealed only subtle or no bone abnormalities, respectively,^16^, ^17^ and (iii) SLC20A1 overexpression in rats did not affect bone mineralization.^18^

By contrast, identification of heterozygous pathogenic variants in *SLC20A2* that cause autosomal dominantly‐inherited primary familial brain calcification (PFBC; idiopathic basal ganglia calcification [IBGC]) indicates a role for SLC20A2 in the regulation of tissue mineralization. PFBC is characterized by extensive brain calcification, predominantly localized to the walls of cerebral microvessels.^19^, ^20^, ^21^ This phenotype is recapitulated in SLC20A2‐deficient mice, in which a local increase in Pi concentrations in the cerebrospinal fluid (CSF) has been suggested to contribute to Ca‐Pi deposition in CSF‐producing tissues and microvessels.^22^, ^23^ Preliminary histomorphometric analysis of SLC20A2‐deficient mice also suggested a role for SLC20A2 in skeletal mineralization,^24^ whereas SLC20A2 expression in vascular smooth muscle has been proposed to inhibit vascular calcification.^25^

To determine the physiological role of SLC20A2 in tissue mineralization, we performed detailed characterization of the skeletal and dental phenotypes of SLC20A2‐deficient mice. These studies identify SLC20A2 as both a physiological regulator of mineralization and an important determinant of bone quality and strength in mice. To determine the physiological role of SLC20A2 in tissue mineralization in humans we also evaluated bone density in PFBC patients.

## Materials and Methods

### Mice

C57BL/6NTac‐*Slc20a2^tm11a(EUCOMM)Wtsi^* (*Slc20a2^+/–^*) heterozygous mice were obtained from the European Mouse Mutant Archive (EMMA) and maintained and genotyped at the Wellcome Trust Sanger Institute (WTSI) or Université de Nantes. Further details about the targeting construct are available at the website for the International Mouse Phenotyping Consortium (IMPC; http://www.mousephenotype.org/data/alleles/MGI:97851/tm1a(EUCOMM)Wtsi). At Université de Nantes, experiments on mice were conducted according to the French and European regulations on care and protection of laboratory animals (EC Directive 86/609, French Law 2001‐486 issued on June 6, 2001) and to the NIH Office of Laboratory Animal Welfare (OLAW; Project #02286.02). Animal care and maintenance were provided through the Université de Nantes accredited animal facility at the “Unité de Thérapeutique Expérimentale.” Mice were fed with RM1 for maintenance and with RM3 for breeding (Special Diets Services [SDS], Essex, UK). To determine intact FGF23, phosphorus, calcium, and creatinine concentrations, mice were weaned with 0.55% Pi and 0.70% Ca diets (Sniff Spezialdiäten GmbH, Soest, Germany). *Slc20a2^–/–^* mice were compared with heterozygous or WT littermates. Mouse studies undertaken by Sanger Mouse Pipelines as part of International Knockout Mouse (IKMC) and IMPC Consortia were licensed by the UK Home Office in accordance with the Animals (Scientific Procedures) Act 1986 and the recommendations of the Weatherall report. All mice generated by Sanger Mouse Pipelines undergo a broad primary phenotype screen (IMPC; http://www.mousephenotype.org) that includes measurement of body length, X‐ray skeletal survey, DXA analysis of BMD, and biochemical measures of mineral metabolism performed between 14 and 16 weeks of age. Mice were fed Mouse Breeder Diet 5021 (Labdiet, London, UK). Pipeline and husbandry details have been reported.^26^, ^27^ Additional WT, *Slc20a2^+/–^*, and *Slc20a2^–/–^* mice were analyzed at postnatal days P1, P16, P18, P21, P28, P35, P49, P98, P112, and P224 (Supporting Fig. S1). Investigators were blinded to genotype during all sample collections and sample processing: genotypes were only assigned to individual samples for final data analysis.

### Serum and urine biochemistry

At P28, serum phosphate and calcium were analyzed using the Phosphorus Liqui‐UV Test and the Calcium CPC LiquiColor Test kits according to the manufacturer's protocol (Stanbio Laboratory, Boerne, TX, USA). Alkaline phosphatase (ALP) enzymatic activity was measured from 2 μL of serum using the ALP Substrate kit (BioRad 172‐1063; Bio‐Rad Laboratories, Hercules, CA, USA). Serum intact FGF23 concentrations were assessed using an ELISA kit according to the manufacturer's protocol (Kainos Laboratories, Tokyo, Japan). At P112, serum phosphate, calcium, and ALP were determined as part of the Sanger Mouse Pipelines primary phenotype screen. Additional urine Pi, Ca, and creatinine assays were performed with Olympus AU400 Chemistry Analyzer and serum intact FGF23 determined using the Kainos Laboratories ELISA kit.

### Skeletal and tooth preparations

Femurs from P1 mice and heads from P112 mice were isolated, fixed in 4% paraformaldehyde for 24 to 48 hours and photographed using a Leica M125 binocular microscope (Leica AG, Wetzlar, Germany) and Leica IC80 HD digital camera. Bone lengths were determined digitally following linear calibration of pixel size.

### X‐Gal staining

Organs dissected from postnatal mice were fixed for 30 to 120 min at room temperature in either 4% paraformaldehyde (PFA) or 2% PFA and 0.2% glutaraldehyde in PBS. During fixation, organs were cut in half after 30 min to improve penetration of fixative and staining solutions. After fixation, samples were rinsed with PBS and incubated overnight at 32°C in X‐Gal staining solution (PBS containing 0.01% Tween 20, 2mM MgCl_2_, 4mM K_4_Fe(CN)_6_, 4mM K_3_Fe(CN)_6_, and 1 mg/mL of X‐gal; Invitrogen, La Jolla, CA, USA). Samples were postfixed in 4% PFA and stored at 4°C. Images were acquired using a M125 stereo‐microscope (Leica) equipped with an IC80HD camera (Leica). For histology, X‐gal–stained skeletal samples were decalcified in 0.5M EDTA (pH 8.0) at 4°C and embedded in paraffin and cut at 7 μm thickness using a Leica RM2255 microtome. Sections were counterstained with nuclear fast red.

### Whole mount stains

P1 mice were euthanized, fixed, and stored in 70% ethanol. Skin and viscera were removed, and the intact skeleton stained with Alizarin red and Alcian blue and stored in 100% glycerol.^28^ Stained P1 mice were imaged using a M125 stereo‐microscope (Leica) equipped with an IC80HD camera (Leica).

### Histology

P21 limbs were fixed in 10% neutral buffered formalin for 24 hours and decalcified in 10% EDTA pH 7.4 for 14 days. 5‐μm paraffin‐embedded sections were stained with Alcian blue and van Gieson.^28^ Images of the proximal tibia were acquired using a Leica DM LB2 microscope and DFC320 digital camera. Measurements at four locations across the width of growth plates were obtained to calculate mean heights of the reserve, proliferative, and hypertrophic zones, and total growth plate. Results from two midline levels of sectioning were compared to ensure data consistency. Reserve, proliferative, and hypertrophic zone chondrocyte numbers were also determined in a 200‐μm‐wide region at the center of the tibial growth plate section using the Cell Counter Plugin in ImageJ 1.46 software (NIH, Bethesda, MD, USA; https://imagej.nih.gov/ij/).

P16 femurs and P28, P112, and P224 heads were fixed in 4% PFA for 24 to 72 hours, dehydrated in graded ethanol, and embedded in methyl methacrylate resin (Technovit^®^ 9100; Kulzer GmbH, Wehrheim, Germany). Five‐micrometer (5‐μm) undecalcified sections were cut using a hard tissue microtome (Leica polycut SM 2500; Leica, Wetzlar, Germany), collected on polylysine‐coated slides and stained with Von Kossa^15^ or Movat's pentachrome. P112 humeri were embedded in poly(methyl methacrylate) (PMMA). Five‐micrometer (5‐μm) midline sagittal sections were cut using a Leica RM2265 microtome and stained with von Kossa/Paragon.

Tibias used for immunohistochemistry (IHC) were fixed in 4% PFA in PBS for 24 hours and decalcified in 0.5M EDTA before embedding in paraffin (Leica TP1020 automated tissue processor). Four‐micrometer (4‐μm) serial sections were cut using a Leica RM2255 microtome. Antigen retrieval conditions and antibodies are reported in Supporting Table S1. Secondary anti‐mouse biotinylated goat antibody (1:500; DAKO, Carpinteria, CA, USA) was used and staining performed using a diaminobenzidine chromogen. Stained sections were mounted with Eukitt^®^ (Dutscher, France) and scanned using a Hamamatsu NanoZoomer HT (Hamamatsu Photonics KK, Hamamatsu City, Japan) digital scanner at magnification ×20.

### Digital X‐ray microradiography

Soft tissue was removed and digital X‐ray images recorded at 10 µm resolution using a Faxitron MX20 point projection X‐ray source and digital image system operating at 26 kV and 5× projective magnification (Qados, Cross Technologies Plc, Sandhurst, UK). Bone mineral content (BMC) was determined relative to steel, aluminum, and polyester standards and median gray levels determined. Images were calibrated with a digital micrometer and bone length determined as described.^28^

### Micro–computed tomography

P112 long bones were analyzed by micro–computed tomography (µCT) (Scanco µCT50; Scanco Medical AG, Brüttisellen, Switzerland) operating at 70 kV, 200 μA, with a 0.5‐mm aluminum filter. Trabecular bone parameters (bone volume [BV/TV], trabecular number [Tb.N], trabecular thickness [Tb.Th]) were determined at 3‐µm resolution in a 1.5‐mm region beginning 100 µm proximal to the distal growth plate. Cortical bone parameters (cortical thickness [Ct.Th], BMD, external cortical diameter) were determined at 10 µm voxel resolution in a 1.5‐mm region centered in the mid‐shaft region 56% along the length of the femur distal to the femoral head. Sixteen‐bit (16‐bit) Digital Imaging and Communications in Medicine (DICOM) images were imported using 32‐bit Drishti v2.0.221 (National Computational Infrastructure, The Australian National University, Acton, ACT, Australia; http://anusf.anu.edu.au/Vizlab/drishti/) and rendered using 64‐bit Drishti v2.0.000 to generate high‐resolution images.

P21 long bones were analyzed using a SkyScan‐1272 X‐ray μCT system (Skyscan, Aartselaar, Belgium), operating at 50 kV peak energy detection and 5 µm pixel resolution. Images were reconstructed using Skyscan NRecon and CTVox softwares. Heads of P28 and P224 WT and Slc20a2^–/–^ mice were analyzed by μCT (Skyscan 1272) at 50 kV and 173 μA with a pixel size of 7 μm. Images were reconstructed using Skyscan NRecon and CTVox software. 3D reconstruction of P49 mice were analyzed by μCT (Skyscan 1072) at 50 kV and 200 μA using a 0.5‐mm aluminum filter and a pixel size of 36 μm.

### Biomechanical testing

Humeri and caudal vertebrae (Ca6 and Ca7) were stored and tested in 70% ethanol. Destructive three‐point bend tests on humeri and compression tests on vertebrae were performed using an Instron 5543 load frame and 100‐N or 500‐N load cells (Instron Limited, High Wycombe, Buckinghamshire, UK). Humeri were positioned on custom supports and load was applied perpendicular to the mid‐diaphysis with a constant rate of displacement of 0.03 mm/s until fracture. Vertebrae were bonded in vertical alignment to a custom anvil support using cyanoacrylate glue and load was applied vertically at a constant rate of displacement of 0.03 mm/s and a sample rate of 20 Hz. Yield load, maximum load, fracture load, and stiffness were determined from load displacement curves.^29^

### Bone quality analysis

Correlation between BMC and biomechanical parameters was determined by linear regression analysis using reference data, updated every month, obtained from femur samples from 320 age‐ and sex‐matched WT mice in an identical genetic background. Femoral BMC (median gray level) correlated with yield load (*p* = 0.005), maximum load (*p* < 0.00001), fracture load (*p* = 0.00003), and stiffness (*p* = 0.003). Bone quality was investigated in female mutant mice by comparing BMC and observed biomechanical parameters in *Slc20a2^+/–^* and *Slc20a2^–/–^* femurs with WT reference data.

### Back‐scattered electron–scanning electron microscopy

Femurs were fixed in 70% ethanol, opened longitudinally and macerated.^28^ Carbon‐coated samples were imaged using back‐scattered electron–scanning electron microscopy (BSE‐SEM) on a Deben Gen5 BSE detector (Deben UK Ltd., Bury St Edmunds, UK) and Tescan VEGA‐3‐XMU scanning electron microscope (Tescan‐UK Ltd., Cambridge, UK). High‐resolution images were quantified using ImageJ to determine the fraction of trabecular and endosteal bone surfaces displaying osteoclastic resorption.^28^

### Quantitative BSE‐SEM

Neutral buffered formalin fixed humeri were embedded in methacrylate. Longitudinal block faces were cut through specimens, which were then polished, coated with carbon, and analyzed using a Deben Gen5 BSE detector and Tescan VEGA‐3‐XMU scanning electron microscope at 20 kV and 0.5 nA with a working distance of 17 mm. BMDs were determined by comparison to halogenated dimethacrylate standards, and an eight‐interval pseudocolor scheme was used to represent the graduations of micromineralization.^28^, ^30^, ^31^

### SEM

Technovit resin‐embedded P28 and P224 head samples were polished on a Metaserv 2000 (Buehler, Lake Bluff, IL, USA) and P28 incisors were cryofractured. Samples were carbon‐coated on a Desk III (Denton Vacuum, Moorestown, NJ, USA) and studied by backscattered SEM (Leo 1450 VP; Zeiss, Oberkochen, Germany), at 20 kV.

### Energy dispersive X‐ray spectroscopy

Energy dispersive X‐ray spectroscopy (EDX) analysis was performed on P16 tibia and incisors and molars from P28 and P224 mice as described.^15^

### Osteoclast histomorphometry

Osteoclast numbers were determined according to the American Society for Bone and Mineral Research (ASBMR) system^31^ in paraffin sections from decalcified tibias stained for tartrate resistant acid phosphatase (TRAP) activity, counterstained with Aniline blue, and imaged using a Leica DMLB2 microscope and DFC320 digital camera.^28^ For each sample, sections from two separate levels (*n* = 2 slides) were photographed at magnification ×100 and a 1‐mm × 1‐mm ROI commencing 250 µm below the growth plate was analyzed. Osteoclast numbers per bone perimeter (Oc.N/B.Pm), osteoclast perimeter (Oc.Pm), and osteoclast surface per bone perimeter (Oc.S/B.Pm) were determined in trabecular bone and normalized to total bone perimeter (B.Pm).^31^ The fraction of bone surfaces that displayed osteoclastic bone resorption was also quantified in high‐resolution BSE‐SEM images.^28^

### Dynamic histomorphometry of bone formation

2D parameters of bone formation were determined according to the ASBMR.^31^ Mice were given intraperitoneal injections of calcein (10 mg/kg in 100 μL PBS) 5 and 3 days prior to euthanasia.^28^ Midline longitudinal block faces were cut through methacrylate‐embedded long bones and calcein labels imaged with a Leica SP5 confocal microscope at 488 nm excitation to determine the fraction of bone surface undergoing active bone formation.^28^ Montages of 20 fields (magnification ×40) were constructed for each bone, and mineralizing surface (MS) and mineral apposition rate (MAR) determined by quantifying calcein‐labeled surfaces and the mean separation between calcein double labels using ImageJ. Bone formation rate (BFR) was calculated by multiplying MS and MAR. Undecalcified proximal humerus sections were also stained with von Kossa/Paragon to determine the osteoid surface deposited as a fraction of total bone perimeter (OS/B.Pm) and the osteoid thickness (O.Th).^32^

### RNA isolation and RT‐qPCR

Total RNA was prepared with TRIzol Reagent (ThermoFisher Scientific, France), followed by Nucleospin RNA columns (Macherey‐Nagel, Düren, Germany) (for calvaria, diaphysis, and ribs) or using only Nucleospin RNA columns (for soft tissues) according to the manufacturer's instructions. Of total RNA, 1 µg was reverse transcribed and analyzed using a Bio‐Rad CFX96 detection system using SYBR Select Master Mix (Applied Biosystems, Warrington, UK). mRNA levels were normalized relative to Tubulin, Glucuronidase, and/or Pinin expression and quantified using the ΔΔCT method.^33^ RT‐qPCR primers were described^34^, ^35^ or are listed in Supporting Table S2.

### Primary chondrocyte and osteoblast cultures

Primary rib chondrocytes were isolated from P7 mice as described^36^ and cultured in Dulbecco's modified Eagle's medium (DMEM) (ThermoFisher Scientific, France) supplemented with 10% FBS. At confluence, cells were plated as described.^37^ Cells were differentiated in DMEM supplemented with 10% FBS, 100 IU/mL penicillin, 100 µg/mL streptomycin, 50 µg/mL of ascorbic acid, and 5mM β‐glycerophosphate. Primary calvarial osteoblasts were isolated from P6 mice and cultured as described.^38^ Cells were differentiated in αMEM (Eurobio, France) supplemented with 10% FBS, 100 IU/mL penicillin, 100 µg/mL streptomycin, 50 µg/mL of ascorbic acid, and 5mM β‐glycerophosphate. Mineralization was determined by Alizarin Red S staining, and the area and size of mineralized nodules were quantified using ImageJ.

### Phosphate uptake measurements

Uptake of phosphate into primary chondrocytes and osteoblasts was determined as described.^35^

### Patients

Patients with *SLC20A2* variants were identified as reported.^20^ Cranial non–contrast‐enhanced CT scans were performed as part of the routine PFBC diagnostic workup and DICOM images were analyzed retrospectively. Age‐ and sex‐matched controls were randomly selected from CT scans performed in the Rouen University Hospital. Bone density was determined in right and left occipital condyle spongiosa and parietal bone thickness was determined 5 cm posterior to the coronal suture.

### Statistics

Power calculations were performed to determine sample sizes based on extensive prior experience and data obtained from our high‐throughput skeletal phenotype analysis^29^ of >700 knockout mouse strains generated by the International Knockout Mouse Consortium (IKMC). We determined coefficients of variation in >250 C57BL6/J WT mice for a comprehensive set of skeletal phenotype parameters as follows: bone length (2%); BMC by X‐ray microradiography (2%); cortical bone parameters by μCT (4% to 6%); trabecular bone parameters by μCT (7% to 15%); bone mineralization by BSE‐SEM (6%); maximum load (9%); and fracture load (20%). Thus, a group size of *n* = 4 is required for 90% power to detect a 20% phenotype difference at a significance level of *p* < 0.05 when using an analytical method with a coefficient of variation of 10%. Data are shown as mean ± SE and were analyzed by ANOVA followed by two‐sided Tukey's post hoc test or unpaired two‐sided Student's *t* test as appropriate; *p* values <0.05 were considered significant. Frequency distributions of mineralization densities obtained by X‐ray microradiography and quantitative BSE (qBSE) were compared using the Kolmogorov‐Smirnov test in which *p* values for the *D* statistic in 1024‐pixel data sets were *D* = >6.01 (*p* < 0.05), *D* = >7.20 (*p* < 0.01), and *D* = >8.62 (*p* < 0.001).

## Results

### Primary phenotype of *Slc20a2^–/–^* knockout mice

**Figure 1:**
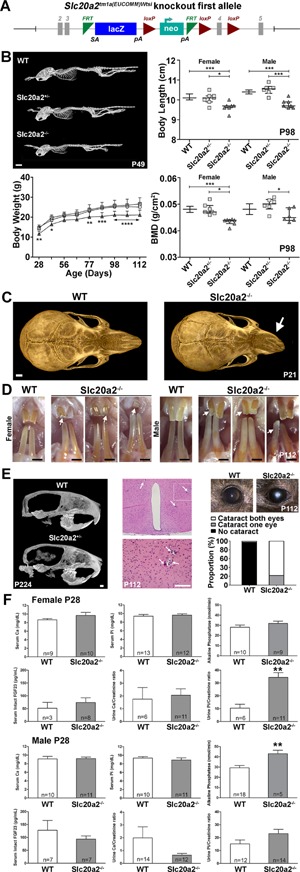
Primary phenotype of *Slc20a2^–/–^* mice. (*A*) *Slc20a2* knockout‐first conditional ready allele (reporter‐tagged insertion allele). The gene‐trap knockout is generated using a targeting cassette containing lacZ and neomycin marker genes. (*B*) μCT images of P49 female WT, *Slc20a2^+/–^*, and *Slc20a2^–/–^* mice. Scale bar = 5 mm. Body length in female and male WT, *Slc20a2^+/–^*, and *Slc20a2^–/–^* mice at P98 (medial with interquartile range, *n* = 6 to 1474 per sex, per genotype, *p < 0.05, ***p < 0.001; Kruskal‐Wallis test followed by Dunn's post hoc test). Body weight in female WT, *Slc20a2^+/–^*, and *Slc20a2^–/–^* mice between P28 and P112 (mean ± SD, *n* = 6 to 34, per genotype per age, *p < 0.05, **p < 0.01, ***p < 0.001, ****p < 0.0001 versus WT; ANOVA followed by Tukey's post hoc test). DXA BMD in female and male WT, *Slc20a2^+/–^*, and *Slc20a2^–/–^* mice at P98 (median with interquartile range, *n* = 6 to 1469 per sex, per genotype, *p < 0.05, ***p < 0.001; Kruskal‐Wallis test followed by Dunn's post hoc test). (*C*) Apical μCT images of skulls from P21 WT and *Slc20a2^–/–^* mice. Scale bar = 1 mm. Arrows indicate abnormal nasal bones in *Slc20a2^–/–^* mice. (*D*) Images of incisors from male and female P112 WT and *Slc20a2^–/–^* mice. Scale bars = 1 mm. (*E*) Midline sagittal μCT images of skulls from WT and *Slc20a2^–/–^* mice at P224. Scale bar = 1 mm. Low‐power and high‐power coronal sections of the brain in *Slc20a2^–/–^* mice at P112; arrows indicate soft tissue calcification. Scale bar = 100 µm. Images of the eye in WT and *Slc20a2^–/–^* mice at P112 and the incidence of cataract. (*F*) Serum and urinary calcium (Ca) and phosphate (Pi) and serum intact FGF23 and ALP activity in female and male WT and *Slc20a2^–/–^* mice at P28 (mean ± SE, *n* = 3 to 14 per genotype per age, **p < 0.01, ***p < 0.001 versus WT; Mann‐Whitney *U* test).

**Figure 2:**
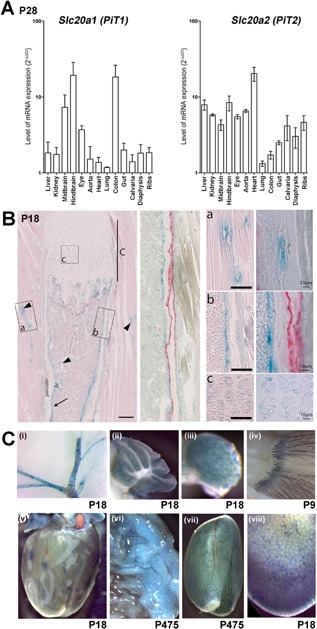
Primary phenotype of *Slc20a2^–/–^* mice. (*A*) Relative *Slc20a1* and *Slc20a2* mRNA expression (mean ± SE) in skeletal and nonskeletal tissues. Levels of *Slc20a1* and *Slc20a2* mRNAs in tissues were determined relative to expression in stomach after normalization to *GusB* and *Tubulin* (*n* = 3). (*B*) LacZ expression from the *Slc20a2^tm11a(EUCOMM)Wtsi^* allele in rib sections from P18 *Slc20a2^–/–^* mice: (a) skeletal muscle blood vessels; (b) cortical bone; and (c) growth plate cartilage. (*C*) Lac Z expression in (i) brain arteries, (ii) cerebellum, (iii) ovary, (iv) hair follicles, (v) heart, (vi) adipose tissue, (vii) testis, and (viii) liver from P18 to P457 *Slc20a2^+/–^* mice.

*Slc20a2^–/–^* knockout mice were generated as part of the IKMC and IMPC at the Wellcome Trust Sanger Institute using a knockout‐first conditional ready targeting vector resulting in mice harboring a reporter‐tagged insertion allele (*Slc20a2^tm11a(EUCOMM)Wtsi^*) (Fig. 1 *A*). A comprehensive summary of the phenotype analysis performed is included in Supporting Fig. S1. Homozygous *Slc20a2^–/–^* knockout mice had reduced body length at postnatal day P98: Females 10.10cm (95% CI, 10.09 to 10.11cm; WT, *n* = 1460), 10.10cm (95% CI, 9.81 to 10.33cm; *Slc20a2^+/–^*, *n* = 7) and 9.64cm*** (95% CI, 9.42cm to 9.86cm; *Slc20a2^–/–^*, *n* = 7) (***p < 0.001 versus WT or *p < 0.05 versus *Slc20a2^+/–^*; Kruskal‐Wallis test). Males 10.40cm (95% CI, 10.37cm to 10.39cm; WT, *n* = 1475), 10.55cm (95% CI, 10.24cm to 10.79cm; *Slc20a2^+/–^*, *n* = 6) and 9.70cm*** (95% CI, 9.60cm to 10.00cm; *Slc20a2^–/–^*, *n* = 7) (***p < 0.001 versus WT or *Slc20a2^+/–^*; Kruskal‐Wallis test). *Slc20a2^–/–^* knockout mice also had reduced body weight at birth: 1.486 ± 0.196g (WT, *n* = 14), 1.490 ± 0.244g (*Slc20a2^+/–^*, *n* = 31), and 1.286 ± 0.166g** (*Slc20a2^–/–^*, *n* = 14) (**p < 0.01 versus WT or *Slc20a2^+/–^*; Mann‐Whitney *U* test) that persisted until P116 (Fig. 1 *B*). Female and male homozygous *Slc20a2^–/–^* mice had low BMD at P98 compared to WT and heterozygous *Slc20a2^+/–^* littermates (Fig. 1 *B*; data not shown). X‐ray microradiography and μCT imaging of P21 and P112 skulls revealed lateral deviation of the nasal bones and maxilla in 33% (8/24) of *Slc20a2^–/–^* mice (Fig. 1 *C*; Supporting Fig. S2), suggesting asymmetric and premature fusion of the skull sutures. Accordingly, quantitative morphometric analysis demonstrated marked decreases in multiple cranial and dental morphological parameters in P112 *Slc20a2^–/–^* mice compared to heterozygous and WT littermates (Supporting Fig. S2). Furthermore, P112 *Slc20a2^–/–^* mice had severe dental fragility was evidenced by frequent incisor fractures (Fig. 1 *D*). At P224, *Slc20a2^–/–^* incisors exhibited an irregular yellow‐brown discoloration and chipping that further evidenced increased fragility. μCT analysis of P224 male and female *Slc20a2^–/–^* mice revealed brain calcification, which was confirmed in histological sections from P112 *Slc20a2^–/–^* mice that revealed calcified lesions within basal ganglia arterioles. Ectopic brain calcification was accompanied by a markedly increased incidence of cataracts in *Slc20a2^–/–^* mice (Fig. 1 *E*). At P28, serum phosphate (Pi), calcium (Ca), and fibroblast growth factor 23 (FGF23) levels were unchanged in *Slc20a2^–/–^* mice (Fig. 1 *F*). The urinary Ca/creatinine ratio was normal in both female and male *Slc20a2^–/–^* mice, whereas female *Slc20a2^–/–^* mice had an increased urinary Pi/creatinine ratio an abnormality that had normalized by P112 (Supporting Fig. S3A). Serum ALP activity was increased in P28 *Slc20a2^–/–^* males (Fig. 1 *F*) and in both males and females at P112 (Supporting Fig. S3A‐B). This increase cannot be explained by abnormal PTH^39^ or vitamin D status (data not shown). *Slc20a1* and *Slc20a2* mRNAs were expressed in similar tissues except for a predominance of *Slc20a1* in colon and cerebellum and *Slc20a2* in the heart. Both *Slc20a1* and *Slc20a2* were expressed in calvaria, long‐bone diaphysis, and rib cartilage (Fig. 2 *A*). LacZ staining of tissues from P18 *Slc20a2^–/–^* mice demonstrated expression in blood vessels, cortical bone osteoblasts, and proliferating and hypertrophic growth plate chondrocytes (Fig. 2 *B*). LacZ staining was also evident in several extraskeletal tissues, including arteries within the brain, cerebellum, ovary, hair follicles, heart, visceral adipose tissue, testis, and liver (Fig. 2 *C*).

In summary, *Slc20a2^–/–^* mice recapitulate the phenotype of symmetric calcification in the basal ganglia seen in PFBC, but also have defective skeletal and dental development together with abnormal tissue mineralization.

### Decreased bone strength and quality in *Slc20a2^–/–^* mice at P112

**Figure 3:**
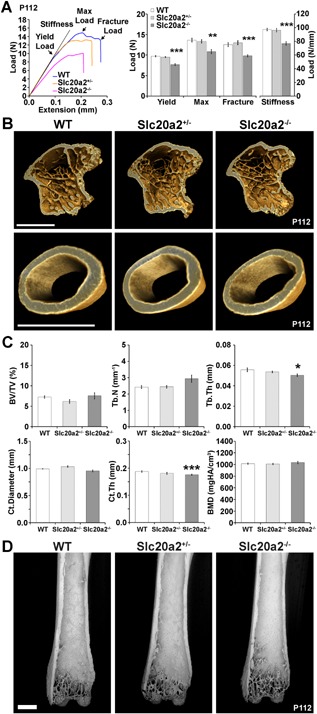
Decreased bone strength and stiffness in *Slc20a2^–/–^* mice. (*A*) Representative load displacement curves from three‐point bend testing of humeri from P112 WT, *Slc20a2^+/–^*, and *Slc20a2^–/–^* female mice showing yield load, maximum load, fracture load, and the gradient of the linear elastic phase (stiffness). Graphs showing yield, maximum load, and fracture load, and stiffness (mean ± SE, *n* = 6, per genotype, **p < 0.01, ***p < 0.001 versus WT; ANOVA followed by Tukey's post hoc test). (*B*) μCT images of the proximal tibial metaphysis and mid‐diaphyseal tibial cortical bone from P112 WT, *Slc20a2^+/–^*, and *Slc20a2^–/–^* female mice. Scale bar = 1 mm. (*C*) Graphs showing BV/TV, Tb.N, Tb.Th, Ct.Diameter, Ct.Th, and cortical BMD (mean ± SE, *n* = 6 per genotype, *p < 0.05, ***p < 0.001, versus WT; ANOVA followed by Tukey's post hoc test). (*D*) Representative BSE‐SEM images of the distal femur from P112 WT, *Slc20a2^+/–^*, and *Slc20a2^–/–^* mice. Scale bars = 1 mm (*n* = 4 to 5 per sex, per genotype). BV/TV = trabecular bone volume per tissue volume; Tb.N = trabecular number; Tb.Th = trabecular thickness; Ct.Diameter = external cortical diameter; Ct.Th = cortical thickness.

**Figure 4:**
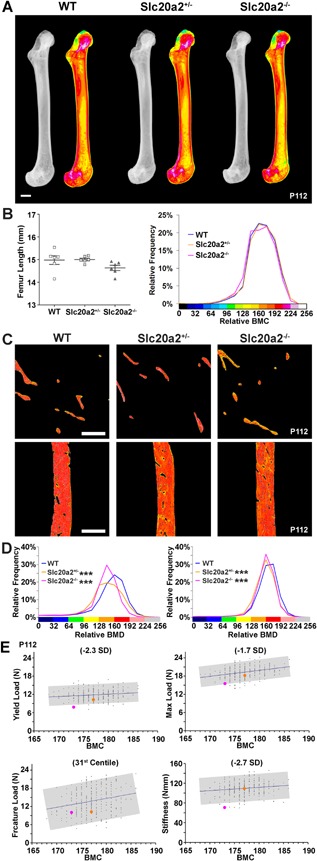
Decreased bone quality and micromineralization density in *Slc20a2^–/–^* mice. (*A*) Quantitative X‐ray microradiography images of femurs from P112 WT, *Slc20a2^+/–^*, and *Slc20a2^–/–^* female mice. Scale bars = 1 mm. Pseudocolored images represent grayscale images using a 16‐color interval scheme with low BMC in blue, and high BMC in red. (*B*) Graph shows femur length (mean ± SE, *n* = 6 per genotype) and relative frequency histogram shows relative BMC. (*C*) Representative quantitative BSE‐SEM images of proximal humerus trabecular bone and humerus cortical bone from P112 WT, *Slc20a2^+/–^*, and *Slc20a2^–/–^* female mice. Scale bars = 250 µm. Grayscale images were pseudocolored using an eight‐color interval scheme with low mineralization density in blue and high mineralization density in red/pink. (*D*) Relative frequency histograms of trabecular (left) and cortical (right) bone micromineralization densities (BMD) (*n* = 4 per genotype, ***p < 0.001 versus WT; Kolmogorov‐Smirnov test). (*E*) Bone quality analysis. Graphs demonstrating the physiological relationship between relative bone mineral content (median gray level determined by quantitative X‐ray microradiography) and yield load, maximum load, fracture load, and stiffness in femurs from P112 female WT mice of identical genetic background (*n* = 320). The line shows the linear regression and the gray box indicates ± 2SD or 95% confidence intervals. Mean values for female heterozygous *Slc20a^+/–^* mice are shown as orange circles and for female homozygous *Slc20a2^–/–^* mice as pink circles; in data from 320 WT, BMC correlated significantly with yield load (*p* = 0.005), maximum load (*p* < 0.00001), fracture load (*p* = 0.00003), and stiffness (*p* = 0.003).

Biomechanical testing demonstrated profoundly decreased bone strength and stiffness in adult *Slc20a2^–/–^* long bones from both sexes (Fig. 3 *A*, Supporting Fig. S4A) and decreased strength in vertebrae from male *Slc20a2^–/–^* mice (Supporting Fig. S5A). However, μCT structural analysis only revealed decreases in some cortical and trabecular bone parameters in adult *Slc20a2^–/–^* mice (Fig. 3 *B*, *C*; Supporting Fig. S4B, C). These observations were confirmed by BSE‐SEM imaging of macerated femurs (Fig. 3 *D*, Supporting Fig. S4D). Furthermore, femur BMC was normal in female *Slc20a2^+/–^* and *Slc20a2^–/–^* mice but decreased in males (Fig. 4 *A*, *B*; Supporting Fig. S6A, B). Vertebral BMC was also decreased in both female and male *Slc20a2^–/–^* mice (Supporting Fig. S5B, D).

Bone composition was thus analyzed by EDX. The Ca/Pi ratio was increased in *Slc20a2^+/–^* and *Slc20a2^–/–^* mice (WT, 1.65 ± 0.04; *Slc20a2^+/–^*, 1.83 ± 0.04*; *Slc20a2^–/–^*, 1.85 ± 0.04**), demonstrating abnormal mineral composition in both heterozygous and homozygous mutants (*p < 0.05, **p < 0.01, *n* = 4 to 7 per genotype, ANOVA followed by Tukey's post hoc test). Consistent with this, quantitative BSE‐SEM (qBSE‐SEM) analysis at a sub‐μm^3^‐resolution scale demonstrated decreased tissue mineralization in trabecular bone from female and male *Slc20a2^+/–^* and *Slc20a2^–/–^* mice, and cortical bone from female *Slc20a2^+/–^* and *Slc20a2^–/–^* mice and male *Slc20a2^–/–^* mice (Fig. 4 *C*, *D*; Supporting Fig. S6C, D). We next investigated bone quality by comparing mineralization and biomechanical parameters in femurs from female *Slc20a2^–/–^* mice with values predicted by linear regression analysis of data from 320 femur samples from age‐ and sex‐matched WT mice on an identical genetic background. Measures of bone quality in *Slc20a2^–/–^* mice were abnormal, with yield load (–2.3 SD), maximum load (–1.7 SD), and stiffness (–2.7 SD) all below WT predicted values (Fig. 4 *E*).

Overall, the profoundly impaired bone strength in adult *Slc20a2^–/–^* mice resulted from abnormalities of bone quality, composition, and mineralization.

### Impaired postnatal skeletal development in *Slc20a2^–/–^* mice

**Figure 5:**
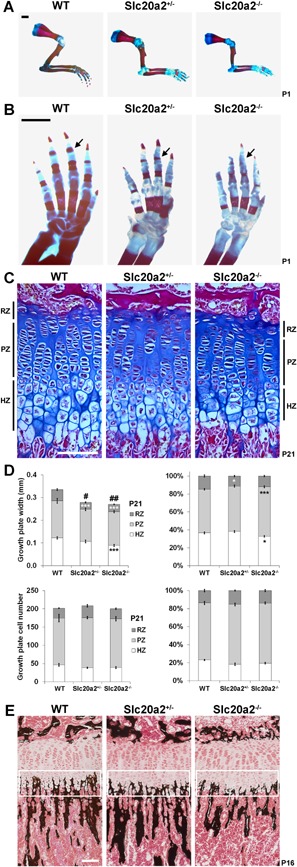
Impaired postnatal skeletal development in *Slc20a2^–/–^* mice. (*A*) Forelimbs from P1 WT, *Slc20a2^+/–^*, and *Slc20a2^–/–^* mice stained with Alizarin red (bone) and Alcian blue (cartilage). Scale bar = 1 mm. (*B*) Forelimb digits stained with Alizarin red and Alcian blue. Scale bar = 1 mm. Black arrows indicate delayed formation of primary ossification centers in P1 *Slc20a2^+/–^* and *Slc20a2^–/–^* mice. (*C*) Decalcified sections of proximal tibia from P21 WT, *Slc20a2^+/–^*, and *Slc20a2^–/–^* mice stained with Alcian blue (cartilage) and van Gieson (bone matrix). Scale bar = 100 µm. (*D*) Upper left graph shows growth plate, RZ, PZ, and HZ heights. Upper right graph shows relative values, where each zone is shown as a percentage of total growth plate height (mean ± SE, *n* = 8 per genotype, *p < 0.05, ***p < 0.001 versus height of zone in WT, #*p* < 0.05, ##*p *< 0.01 versus total growth plate height in WT; ANOVA followed by Tukey's post hoc test). Lower left graph shows chondrocyte cell number in RZ, PZ, and HZ. Lower right shows relative values, where cell number in each zone is shown as a percentage of total cell number (mean ± SEM, *n* = 8 per genotype). (*E*) Undecalcified sections of P16 proximal tibia stained with Von Kossa (mineral deposition in black). HZ ROI is indicated by white rectangle. Scale bar = 100 µm. RZ = reserve zone; PZ = proliferating zone; HZ = hypertrophic zone.

We first investigated skeletal development to determine the mechanisms underlying the abnormalities of bone strength, mineralization, and quality. *Slc20a2^–/–^* mice had delayed formation of primary ossification centers compared to WT and *Slc20a2^+/–^* littermates at P1 (Fig. 5 *A*, *B*) followed by impaired linear growth (Supporting Figs. S5B and D, S6B, and S7A and B). The effects of *Slc20a2* deletion on endochondral ossification were investigated by histological analysis of the proximal tibia growth plate. A 17% reduction in growth plate width, predominantly affecting the reserve and hypertrophic zones, was observed in P21 *Slc20a2^–/–^* mice (Fig. 5 *C*, *D*). Nevertheless, numbers of cells in each zone were similar among WT, *Slc20a2^+/–^* and *Slc20a2^–/–^* mice, suggesting that chondrocyte volume and hypertrophic chondrocyte enlargement are impaired in the absence of Slc20a2 (Fig. 5 *D*). Immunohistochemical staining of type II and type X collagen confirmed the reduced width of the *Slc20a2^–/–^* growth plate but demonstrated similar expression patterns among the genotypes (Supporting Fig. S7C). Furthermore, no differences in expression of osteoblast and osteocyte marker genes or genes involved in mineralization and Pi transport were observed in *Slc20a2^–/–^* mice compared to WT (Supporting Fig. S7D). Nevertheless, Von Kossa staining of proximal tibia sections from *Slc20a2^–/–^* mice showed a 20% decrease in cartilage mineralization compared to WT and heterozygous littermates (Fig. 5 *E*). Consistent with this, juvenile *Slc20a2^–/–^* mice had decreased BMC in long bones and vertebrae and decreases in trabecular and cortical bone parameters compared to WT and heterozygous mutants (Supporting Fig. S7A, B; Supporting Table S3).

Overall, *Slc20a2^–/–^* mice exhibit defective endochondral ossification resulting in impaired linear growth and skeletal mineralization.

### Impaired tooth development and mineralization in *Slc20a2^–/–^* mice

**Figure 6:**
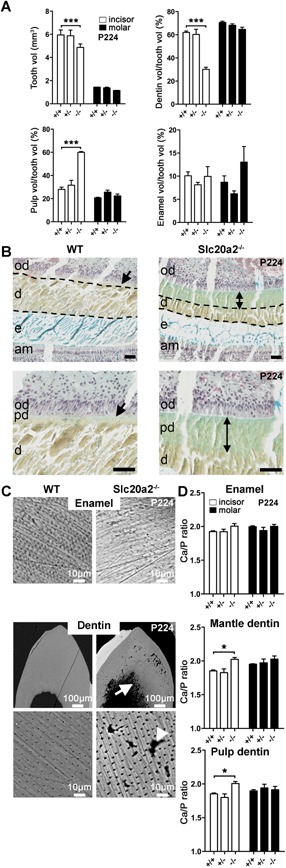
Impaired tooth development and mineralization in *Slc20a2^–/–^* mice. (*A*) Incisor (white bars) and first molar (black bars) μCT parameters from P224 WT, *Slc20a2^+/–^*, and *Slc20a2^–/–^* male mice. Graphs showing total tooth (Tooth vol), pulp (Pulp vol/tooth vol), dentin (Dentin vol/tooth vol), and enamel (Enamel vol/tooth vol) volumes relative to total tooth volume (mean ± SEM, *n* = 3 per genotype, ***p < 0.0001, versus WT; two‐way ANOVA followed by Bonferroni post hoc test). (*B*) Low and higher power images of sagittal sections of P224 mandibular incisors from WT and *Slc20a2^–/–^* male mice stained with Movat. Scale bars = 50 μm. Double arrows indicate increased predentin thickness in *Slc20a2^–/–^* mice. Dotted lines indicate decreased dentine thickness in *Slc20a2^–/–^* mice. (am = ameloblasts, e = enamel, d = dentin, od = odontoblasts, pd = predentin). (*C*) SEM images of enamel and dentin in resin‐embedded mandibular incisors form P224 WT and *Slc20a2^–/–^* male mice. Scale bars = 10 to 100 μm. White arrow indicates calcospherites in incisors of *Slc20a2^–/–^* mice. White arrowhead indicates interglobular spaces in incisors of *Slc20a2^–/–^* mice. (*D*) EDX analysis of incisors (white bars) and first molars (black bars) from P224 WT, *Slc20a2^+/–^*, and *Slc20a2^–/–^* male mice. Graphs showing calcium:phosphate ratio (Ca/P) in enamel (upper), mantle dentin (middle), and circumpulpal (Pulp) dentin (lower) (mean ± SE, *n* = 3 per genotype, *p < 0.01; versus WT; two‐way ANOVA followed by Bonferroni post hoc test). EDX = energy‐dispersive X‐ray spectroscopy.

Because of the high incidence of incisor fracture, we investigated dental mineralization in juvenile and adult *Slc20a2^–/–^* mice. μCT analysis demonstrated decreased incisor and molar volumes in P28 *Slc20a2^–/–^* mice. Dentin and enamel volumes were also reduced, and accompanied by an increased pulp volume (Supporting Fig. S8A). Similar abnormalities were also present in incisors from P224 mutants, likely due to the continuous growth of rodent incisors (Fig. 6 *A*). Movat's pentachrome staining demonstrated normal organization of odontoblast and ameloblast palisades in *Slc20a2^–/–^* mice (Fig. 6 *B*; Supporting Fig. S8B). Nevertheless, there was a marked increase in the unmineralized predentin layer and reduction of the mineralized dentin layer in *Slc20a2^–/–^* mice, indicating a mineralization defect consistent with the increased pulp volume. SEM analysis demonstrated no differences in enamel structure in juvenile and adult *Slc20a2^–/–^* mice. By contrast and consistent with our recent histological studies,^40^ dentin matrix morphology was abnormal with irregular intertubular dentin, unfused calcospherites, and increased interglobular spaces (Fig. 6 *C*, Supporting Fig. S8C). Finally, we determined enamel and dentin composition by EDX. Consistent with the mineral composition abnormalities identified in bone, the Ca/Pi ratio in *Slc20a2^–/–^* mice was also increased in incisor enamel and mantle dentin at P28, and in incisor mantle and circumpulpal dentin at P224 (Fig. 6 *D*, Supporting Fig. S8D).

Thus, even though tooth mineralization progresses in *Slc20a2*‐deficient mice, dentin mineralization and morphology remain defective and teeth frequently fracture.

### Bone resorption and formation in *Slc20a2^–/–^* mice

We next investigated osteoclastic bone resorption and osteoblastic bone formation to determine whether defects in bone strength, mineralization, and quality resulted from abnormal bone turnover. Histomorphometric analysis of osteoclast parameters in proximal tibia sections and determination of endosteal bone resorption by BSE‐SEM demonstrated no differences among WT, *Slc20a2^+/–^*, and *Slc20a2^–/–^* mice (Supporting Fig. S9A). Similarly, dynamic histomorphometry of bone formation in calcein double‐labeled specimens revealed no differences between WT, *Slc20a2^+/–^*, and *Slc20a2^–/–^* mice (Supporting Fig. S9B, C), and these findings were supported by similar osteoid surfaces and thickness in all genotypes (Supporting Fig. S9D).

**Figure 7:**
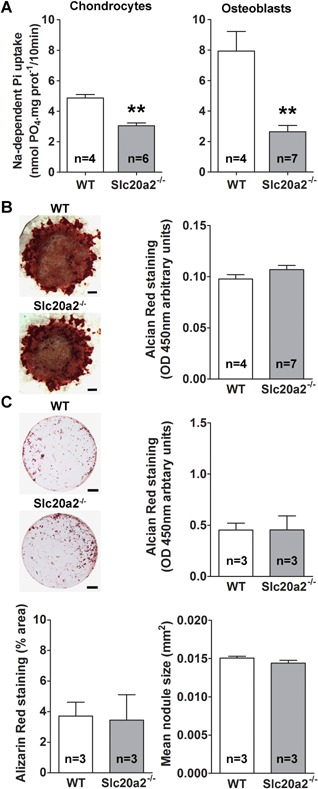
Primary chondrocyte and osteoblast cultures. (*A*) Sodium‐dependent phosphate uptake in primary rib chondrocytes (left) or calvarial osteoblasts (right) from WT and *Slc20a2^–/–^* mice (mean ± SE, *n* = 4 to 7 per genotype, **p < 0.01; versus WT; Mann‐Whitney *U* test). (*B*) Representative images of Alizarin red–stained high‐density chondrocyte pellets from P7 WT and *Slc20a2^–/–^* mice showing mineral deposition after 10 days in culture. Scale bars = 500 µm (*n* = 4 to 7 per genotype). Graph shows quantitation of eluted Alizarin red stain. (*C*) Representative images of Alizarin red–stained colony‐forming calvarial osteoblasts from P6 WT and *Slc20a2^–/–^* mice showing mineral deposition after 29 days in culture. Scale bars = 500 mm (*n* = 3 per genotype). Graphs show quantitation of eluted Alizarin red, fractional area of Alizarin red staining, and mean size of mineralized nodules (mean ± SE, *n* = 3 per genotype; Mann‐Whitney *U* test).

We performed primary cell cultures to determine whether the growth and mineralization abnormalities in *Slc20a2^–/–^* mice resulted from intrinsic defects of chondrocyte or osteoblast function. Absence of *Slc20a2* mRNA expression was verified in *Slc20a2^–/–^* cells by RT‐qPCR a reduction of approximately 50% was demonstrated in heterozygous cells (Supporting Fig. S10A, B), confirming the *Slc20a2^tm11a(EUCOMM)Wtsi^* allele is null. As a result, Na^+^‐Pi transport was reduced by 25% in Slc20a2‐deficient chondrocytes and by 75% in osteoblasts (Fig. 7 *A*). Despite this, no differences in expression of mature chondrocyte markers (*Col10a1*, *Runx2*, *Mmp13*), mineralization regulatory genes (*Alpl*, *Phospho1*) or the phosphate exporter *Xpr1* were observed (Supporting Fig. S10A) and in vitro mineralization of primary chondrocytes from *Slc20a2^–/–^* mice did not differ from WT (Fig. 7 *B*). Similarly, no differences were observed during in vitro differentiation (Supporting Fig. S10B), nodule formation, and mineralization of primary calvarial osteoblasts from *Slc20a2^–/–^* mice (Fig. 7 *C*).

These data demonstrate that *Slc20a2* is essential for normal phosphate transport, whereas chondrocyte and osteoblast differentiation and mineralization were unaffected by deletion of *Slc20a2* in primary cultured cells.

### Cranial μCT analysis of PFBC patients

**Figure 8:**
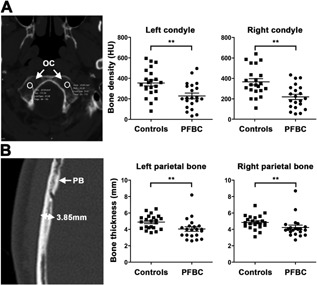
Cranial μCT analysis of PFBC patients and normal control individuals. (*A*) Bone density measurements in left and right OCs from PFBC patients (*n* = 21) and matched controls (*n* = 22). (mean ± SE, Student's *t* test, **p ≤ 0.01). (*B*) Left and right PB thickness measurements in PFBC patients (*n* = 21) and matched controls (*n* = 22). (mean ± SE, Student's *t* test, **p ≤ 0.01). OC = occipital condyle; PB = parietal bone.

Following the identification of major skeletal abnormalities in *Slc20a2^–/–^* mice, we investigated PFBC patients with heterozygous pathogenic variants in *SLC20A2*. A retrospective analysis of medical records from a French series of PFBC patients^20^ revealed none of 24 had cataracts and 4 of 4 had normal serum ALP concentrations. Average height was normal (175.2 cm in males *n* = 10; 164.0 cm in females *n* = 5), although one female had short stature (149.5 cm; –2.5 SD compared to sex‐specific normative values). No data were available regarding tooth mineralization. One individual had suffered a wrist fracture and two postmenopausal women had osteopenia. Bone density and thickness in occipital condyle and parietal bone spongiosa were determined in 21 patients and age‐ and sex‐matched controls using available CT data. Mean bone density and thickness were decreased in PFBC patients compared to controls (Fig. 8 *A*, *B*).

Thus, preliminary analysis of PFBC patients with heterozygous mutations in *SLC20A2* suggests they may also have reduced bone mass and mineralization.

## Discussion

These studies show that *Slc20a2* knockout mice have a severely abnormal skeletal phenotype characterized by a disproportionate reduction in bone strength that cannot be accounted for by the modest reductions in cortical thickness and BMC. Thus, SLC20A2‐deficiency results in an important functional impairment of bone quality characterized by an increased calcium/phosphate ratio. Bone quality represents a composite of interdependent factors that include both structural and material parameters such as: bone geometry; bone microarchitecture; the orientation and interrelationship of matrix proteins and collagen fibrils; and hydroxyapatite crystal size and packing, and has only recently been appreciated as a major contributor to fracture risk.^2^, ^41^, ^42^ Here, we identify *Slc20a2* as a new and important genetic determinant of bone quality.

*Slc20a2* knockout mice display a diverse disorder affecting the skeleton, teeth, brain vasculature, and lenses that is due to abnormal and/or inappropriate tissue mineralization. Extraskeletal abnormalities in *Slc20a2* knockout mice recapitulate the autosomal dominant human condition of PFBC characterized by symmetric calcification in the basal ganglia and other brain regions in affected heterozygous individuals. However, the major dental, skeletal, and ocular abnormalities reported here were not recognized previously^24^ and result from complete SLC20A2 deficiency. Thus, SLC20A2 deficiency causes abnormalities of endochondral and intramembranous ossification that affect skeletal development, linear growth, and mineral accrual, resulting in profound and discordant decreases in bone strength and stiffness. Despite this, in adults, SLC20A2 deficiency leads to only limited effects on trabecular and cortical bone structure, turnover and mineralization density.

### Role of SLC20A2 in the determination of bone quality

In the MV‐based model of mineralization, the nucleation of apatite crystals from Ca and Pi ions occurs within the MV. In this process Pi is provided from both intravesicular and extravesicular sources.^14^ The intravesicular Pi is generated by PHOSPHO1, whereas the extravesicular Pi is thought to be generated by tissue‐nonspecific alkaline phosphatase (TNAP) and transported into the MV via a Pi transporter that is yet to be identified. Because SLC20A1 was shown to be regulated by multiple osteogenic factors and to regulate proliferation, differentiation and mineralization of chondrocytes or osteoblasts in vitro, it was long thought to be the responsible Pi transporter. However, and consistent with the absence of a mineralization defect of *Slc20a1* hypomorphic mice,^15^ we showed that mineralization defects observed in the absence of both PHOSPHO1 and SLC20A1 were not as severe as abnormalities seen in the absence of PHOSPHO1 and TNAP, further illustrating the minor role of Slc20a1 in matrix mineralization.^16^ Although it remains possible that SLC20A2 compensated for decreased expression of SLC20A1 in these studies, the current studies suggest that apatite crystal formation or nucleation still occurs in the absence of SLC20A2. Current data are thus not consistent with an essential role for SLC20A2 in providing Pi for initiation of the mineralization process. Furthermore, because of the emerging role for SLC20A1 in the skeleton and absence of increased *Slc20a1* expression in *Slc20a2*‐deficient cells it is unlikely that mineralization occurring in *Slc20a2*‐deficient cells results from functional compensation by SLC20A1. Nevertheless, functional compensation between SLC20A1 and SLC20A2 and a role for SLC20A2‐mediated Pi transport, in determining dental and bone material properties and quality require further detailed investigation.

#### Systemic effects

The normal serum calcium, phosphate, FGF23, PTH, and 1,25(OH) vitamin D^39^ in *Slc20a2^–/–^* mice preclude a role for SLC20A2 in systemic calcium and phosphate homeostasis. Nevertheless, *Slc20a2* knockout mice display placental dysfunction,^43^ which may contribute to delayed intrauterine endochondral ossification and growth. This important developmental phenotype could be further investigated by conditional deletion of *Slc20a2* using *Mox2‐Cre* or *Sox2‐Cre* mice.^44^, ^45^

#### Growth plate and bone matrix effects

Similarly, systemic phosphate homeostasis cannot account for the skeletal phenotype in *Slc20a2^–/–^* mice, indicating a critical and local cell‐intrinsic role for SLC20A2 in growth plate chondrocytes and osteoblasts that is essential for linear growth and the normal material properties of bone. Furthermore, the current studies demonstrate that *Slc20a2^–/–^*global knockout mice have a contrasting skeletal phenotype to that recently described in mice with inducible chondrocyte‐specific deletion of *Slc20a1* (*Slc20a1^ΔAgc1/ΔAgc1^*).^17^ In *Slc20a1^ΔAgc1/ΔAgc1^* mice, growth plate chondrocytes exhibited massive cell death and endoplasmic reticulum (ER) stress whereas, in *Slc20a2^–/–^* mice, growth plate chondrocytes are reduced in size but not number resulting in impaired linear growth with no evidence of cell death. By contrast, no bone abnormalities were observed in *Slc20a1^ΔAgc1/ΔAgc1^* mice, whereas *Slc20a2^–/–^* global knockout mice have impaired bone strength and bone quality. Thus, chondrocyte‐specific deletion of *Slc20a2* could be used to determine the consequences of SLC20A2 deficiency in the growth plate on adult bone material properties and strength.

#### Dental effects

Systemic phosphate homeostasis cannot explain the dental phenotype in *Slc20a2^–/–^* mice, thus, also indicating a critical and local cell‐intrinsic role for SLC20A2 in sub‐odontoblastic cells.^40^ Importantly, we recently showed that *Slc20a2* is also expressed in lining sub‐odontoblastic cells rather than in mineralizing odontoblasts or ameloblasts, suggesting that SLC20A2 has a role in mediating local crosstalk between mineralizing and nonmineralizing cells that may involve functions other than Pi transport.^40^ Consistent with this, we have also shown that, independently of its Pi transport function, SLC20A2 acts as a Pi‐sensor binding extracellular Pi and activating the ERK1/2 MAPK signaling pathway and FGF23 secretion.^39^, ^46^ Therefore, dental and skeletal mineralization and strength may involve a paracrine role for SLC20A2 or an endocrine role in precisely regulating the phosphate set point that is dependent on its Pi sensing capacity, rather than Pi transport function.

Overall, our current data indicate that SLC20A1 and SLC20A2 possess common roles as phosphate transporters but exert specific and non‐redundant functions in cartilage and bone that cannot be compensated for by each other. These studies further suggest an important cell intrinsic role for SLC20A2 in the skeleton.

### SLC20A deficiency in humans

Preliminary analysis of available μCT data from a cohort of PFBC patients identified abnormalities of cranial bone density and thickness, and these findings suggest that other skeletal and dental abnormalities should be investigated. Furthermore, findings of reduced bone mineralization in heterozygous *Slc20a2^+/–^* mice (Fig. 4, Supporting Fig. S6) and a trend for a gene dose effect on bone strength (Fig. 3, Supporting Figs. S4 and S5) suggest that careful consideration of fracture risk is required in PFBC patients as it may be greater than that predicted by BMD assessment given the profound effect on bone quality observed in homozygous *Slc20a2^–/–^* mice (Fig. 4). Nevertheless, future prospective studies that include (i) standard DXA bone density measurements and (ii) multivariate analysis of Fracture Risk Assessment Tool (FRAX) risk factors will be required to establish the relationship between SLC20A2 deficiency and BMD in PFBC patients.

Currently BMD is used as the key predictor of clinical fracture risk but it fails to take account of bone quality (https://www.sheffield.ac.uk/FRAX/). Accordingly, SLC20A2 was not identified as an independent locus associated with BMD in the largest genomewide association study (GWAS) to date.^47^ Importantly, this GWAS only explains 12% of the BMD phenotype variance and is unable to identify genetic variants with predominant effects on bone quality. Nevertheless, the homologous skeletal Pi transporter *SLC20A1* (*PiT1*) has recently been identified as the most significant candidate gene in a GWAS of clinical vertebral fractures independent of bone density,^48^ further suggesting a critical role for phosphate transport in the determination of bone quality.

### New approaches for bone quality analysis

The challenge for the future will be to develop standards for the assessment of bone quality. This will require validation of novel methods that correlate in vitro and in vivo measures of bone microarchitecture with bone strength and ductility. The IMPC is generating knockout mice for all 20,000 protein encoding genes on an identical genetic background and provides a unique resource to facilitate identification of genetic determinants of bone mass and strength^29^, ^47^ (Origins of Bone and Cartilage Disease Project, London, UK; http://www.boneandcartilage.com/). Here we extend this analysis to identify defects of bone quality for the first time (Fig. 4). To understand the molecular basis for abnormalities of bone quality, our novel approach can now be combined with analyses that determine the microstructural consequences of gene deletion. Such methods will include Fourier transform infrared microspectroscopy (FTIR),^49^ multinuclear solid‐state nuclear magnetic resonance (NMR) spectroscopy, and powder X‐ray diffraction (XRD).^50^

In summary, we have shown that SLC20A2 deficiency results in impaired bone quality and strength. Identification of further genetic determinants of bone quality has the potential to lead to a paradigm shift in bone biology. This will have implications for the clinical assessment of fracture risk and for development of novel therapeutic approaches that complement current drugs, and which target bone turnover.

## Disclosures

All authors state that they have no conflicts of interest.

## Supporting information

Supporting Figure Legends.

Supporting Figure S1.

Supporting Figure S2.

Supporting Figure S3.

Supporting Figure S4.

Supporting Figure S5.

Supporting Figure S6.

Supporting Figure S7.

Supporting Figure S8.

Supporting Figure S9.

Supporting Figure S10.

Supporting Table S1.

Supporting Table S2.

Supporting Table S3.
