# Supplementary material for: Slc20a2, Encoding the Phosphate Transporter PiT2, Is an Important Genetic Determinant of Bone Quality and Strength
Source: J Bone Miner Res. 2019 Mar 19;34(6):1101–14. doi: 10.1002/jbmr.3691 (PMC6618161; doi:10.1002/jbmr.3691)
Supplement: Supplementary file 1 — Supporting Figure Legends. [file JBMR-34-1101-s001.docx]

**Supporting Figure Legends**

**Supporting Fig. S1: Analysis timeline for *Slc20a2^-/-^* mice.**

Timeline summary indicating the postnatal days at which samples were collected for each analytical method and the figure panel in which the data are included. Abbreviations: BMC, Bone mineral content; EDX, Energy Dispersive X-Ray Spectroscopy; BSE-SEM, back-scattered electron-scanning electron microscopy; qBSE-SEM, quantitative back-scattered electron-scanning electron microscopy; DXA BMD, Dual-energy X-ray absorptiometry bone mineral density.

**Supporting Fig. S2: Skull morphometry of *Slc20a2^-/-^* mice.**

**A.** Apical and lateral X-ray microradiographic images of skulls from P21, WT and *Slc20a2^-/-^* mice, scale bar = 1mm.

**B.** Lateral and anterior micro-CT images of skulls from P21 WT and *Slc20a2^-/-^* mice, scale bar = 1mm. Arrows indicate abnormal nasal bones in *Slc20a2^-/-^* mice.

**C.** Morphometric measurements determined in apical and lateral skull microradiographs.

**D.** Graphs showing morphometric parameters (cephalic index = [cranial width/cranial length] x 100) in skulls from P112 WT, *Slc20a2^+/-^* and *Slc20a2^-/-^* mice (mean ± SEM, n=6 per sex, per genotype, **P*<0.05, ***P*<0.01, ****P*<0.001, versus WT; ANOVA followed by Tukey’s post hoc test).

**Supporting Fig. S3: Serum and urine biochemistry in *Slc20a2^-/-^* mice at P112.**

**A.** Serum and urinary calcium (Ca) and phosphate (Pi) and serum intact FGF23 and ALP activity in female WT, *Slc20a^+/-^* and *Slc20a2^-/-^* mice at P112 (mean ± SEM, n=5-27 per genotype per age, *****P*<0.0001; Mann- Whitney U test)

**B.** Serum and urinary calcium (Ca) and phosphate (Pi) and serum intact FGF23 and ALP activity in male WT, *Slc20a^+/-^* and *Slc20a2^-/-^* mice at P112 (mean ± SEM, n=5-35 per genotype per age, ****P*<0.001, *****P*<0.0001; Mann- Whitney U test)

**Supporting Fig. S4: Decreased bone strength and stiffness in male *Slc20a2^-/-^* mice.**

**A.** Representative load displacement curves from 3-point bend testing of humeri from P112 WT, *Slc20a2^+/-^* and *Slc20a2^-/-^* male mice showing yield load, maximum load, fracture load, and the gradient of the linear elastic phase (stiffness). Graphs showing yield, maximum, and fracture loads and stiffness (mean ± SEM, n=6 per genotype, **P*<0.05, ***P*<0.01, versus WT; ANOVA followed by Tukey’s post hoc test).

**B.** Micro-CT images of the proximal tibial metaphysis and mid-diaphyseal tibial cortical bone from P112 WT, *Slc20a2^+/-^* and *Slc20a2^-/-^* female mice, scale bar = 1mm.

**C.** Graphs showing, trabecular bone volume per tissue volume (BV/TV), trabecular number (Tb.N), trabecular thickness (Tb.Th), external cortical diameter (Ct.Diameter), cortical thickness (Ct.Th) and cortical bone mineral density (BMD) (mean ± SEM, n=6, per genotype, **P*<0.05, versus WT; ANOVA followed by Tukey’s post hoc test).

**D**. Representative BSE-SEM images of the distal femur from P112 *WT, Slc20a2^+/-^ and Slc20a2^-/-^* mice, scale bars = 1mm (n=4-5 per sex, per genotype).

**Supporting Fig. S5: Decreased bone mineral content and strength in *Slc20a2^-/-^* mice.**

**A.** Representative load displacement curves from compression testing of proximal caudal vertebrae from P112 WT, *Slc20a2^+/-^* and *Slc20a2^-/-^* male mice showing yield load, maximum load, and stiffness. Graphs showing yield and maximum loads and stiffness (mean ± SEM, n=6 per genotype, **P*<0.05, ***P*<0.01, ****P*<0.001 versus WT; ANOVA followed by Tukey’s post hoc test).

**B.** Quantitative X-ray microradiographic images of vertebrae from P112, WT, *Slc20a2^+/-^* and *Slc20a2^-/-^* male mice, scale bars = 1mm. Pseudocolored images represent gray scale images using a 16-color interval scheme with low bone mineral content (BMC) blue and high mineral content red. Graphs showing, vertebral length (mean ± SEM n=6 per genotype, **P*<0.05, versus WT; ANOVA followed by Tukey’s post hoc test). Relative frequency histograms of bone mineral content (n=6 per sex, per genotype, ***P*<0.01, versus WT; Kolmogorov-Smirnov test).

**C.** Representative load displacement curves from P112 WT, *Slc20a2^+/-^* and *Slc20a2^-/-^* female mice showing (mean ± SEM, n=6 per genotype).

**D.** Quantitative X-ray microradiographic images of vertebrae from P112, WT, *Slc20a2^+/-^* and *Slc20a2^-/-^* female mice. Graphs showing, vertebral length (mean ± SEM, n=6 per genotype, **P*<0.05, ***P*<0.01; ANOVA followed by Tukey’s post hoc test). Relative frequency histograms of relative BMC (n=6 per genotype, ****P*<0.001 versus WT; Kolmogorov-Smirnov test).

**Supporting Fig. S6: Decreased bone mineral content and micromineralization in male *Slc20a2^-/-^* mice**

**A.** Quantitative X-ray microradiography images of femurs from P112, WT, *Slc20a2^+/-^* and *Slc20a^-/-^* male mice, scale bars = 1mm.

**B.** Graph shows femur length (mean ± SEM, n=6 per genotype, **P*<0.05 versus WT; ANOVA followed by Tukey’s post hoc test) and relative frequency histogram shows bone mineral content (BMC) (n=6 per genotype, ****P*<0.001 versus WT; Kolmogorov-Smirnov test).

**C**. Representative quantitative BSE-SEM images of proximal humerus trabecular bone and humerus cortical bone from P112 WT, *Slc20a2^+/-^* and *Slc20a2^-/-^* male mice, bars = 250μm. Relative frequency histogram of trabecular bone micromineralization densities (BMD). Gray-scale images were pseudocolored using an 8-color interval scheme with low mineralization density in blue and high density in red/pink.

**D.** Relative frequency histograms of trabecular (left) and cortical (right) bone micromineralization densities (BMD) (n=4, per genotype, **P*<0.05, ****P*<0.001 versus WT; Kolmogorov-Smirnov test).

**Supporting Fig. S7: Impaired post-natal skeletal development in *Slc20a2^-/-^* mice.**

**A.** Quantitative X-ray microradiography images of femurs from P21, WT, *Slc20a2^+/-^* and *Slc20a2^-/-^* mice, scale bars = 1mm. Pseudocolored images represent gray scale images using a 16-color interval scheme with low bone mineral content (BMC) blue and high BMC red. Graph shows femur length (mean ± SEM, n=6 per genotype, **P*<0.05, ***P*<0.01; ANOVA followed by Tukey’s post hoc test) and relative frequency histogram shows bone mineral content (n=6 per genotype, ****P*<0.001 versus WT; Kolmogorov-Smirnov test).

**B.** Quantitative X-ray microradiographic images of vertebrae from P21, WT, *Slc20a2^+/-^* and *Slc20a2^-/-^* mice, scale bars = 1mm. Graphs showing, vertebral length (mean ± SEM, n=6 per sex, per genotype, **P*<0.05 between WT, *Slc20a2^+/-^* and *Slc20a2^-/-^* genotypes; ANOVA). Relative frequency histogram of BMC (n=6 per sex, per genotype, ****P*<0.001 versus WT; Kolmogorov-Smirnov test).

**C.** Type II collagen and type X collagen expression in decalcified sections of proximal tibia from P35 WT and *Slc20a2^-/-^* mice (representative sections from n=6 per genotype). HZ regions defined by collagen X staining are indicated by two dashed lines. Scale bar: 100µm.

**D.** RT-qPCR analysis of genes involved in differentiation (*Col1a1*, *Ocn*, *Bsp*, *Spp1* and *Dmp1*), mineralization (*Phospho1*, *Enpp1* and *Alpl*) and phosphate transport (*Xpr1* and *Slc20a1*) in tibia from P21-35 WT and *Slc20a2^-/-^* mice (mean ± SEM, n=9-10 per genotype).

**Supporting Fig. S8: Impaired tooth development and mineralization in *Slc20a2^-/-^* mice.**

**A.** Incisor (white bars) and first molar (black bars) micro-CT parameters from P28 WT, *Slc20a2^+/-^* and *Slc20a2^-/-^* male mice. Graphs showing total tooth (Tooth vol), pulp (Pulp vol/tooth vol), dentin (Dentin vol/tooth vol) and enamel (Enamel vol/tooth vol) volumes relative to total tooth volume (mean ± SEM, n=6 per genotype, **P*<0.01; ***P*<0.001; ****P*<0.0001, versus WT; 2 way ANOVA followed by Bonferroni post hoc test).

**B.** Low and higher power images of sagittal sections of P28 mandibular incisors from WT and *Slc20a2^-/-^* male mice stained with Movat, Scale bars: 50µm. Double arrows indicate increased predentin thickness in *Slc20a2^-/-^* mice. Dotted lines indicate decreased dentine thickness in *Slc20a2^-/-^* mice. (ameloblasts (am), enamel (e), dentin (d), odontoblasts (od), predentin (pd)).

**C.** SEM images of cryofractured mandibular incisors form P28 WT and Slc20a2^-/-^ male mice, scale bars 2-10µm. Dentin tubule (DT), intertubular dentin (IT).

**D.** Energy Dispersive X-Ray Spectroscopy (EDX) analysis of incisors (white bars) and first molars (black bars) from P28 WT, *Slc20a2^+/-^* and *Slc20a2^-/-^* male mice. Graphs showing, calcium:phosphate ratio (Ca/P) in enamel (upper), mantle dentin (middle) and circumpulpar (Pulp) dentin (lower) (mean ± SEM, n=4-6 per genotype, **P*<0.01; versus WT; 2 way ANOVA followed by Bonferroni post hoc test).

**Supporting Fig. S9: Bone resorption and formation in *Slc20a2^-/-^* mice**

**A.** BSE-SEM images of mid-femur endocortical bone surface from P112 WT, *Slc20a2^+/-^* and *Slc20a2^-/-^* mice, (arrows indicate borders between regions of osteoclastic resorption and unresorbed bone surfaces, bars = 250μm). Decalcified proximal tibia sections from P112 WT, *Slc20a2^+/-^* and *Slc20a2^-/-^* mice stained with TRAP for osteoclasts in red, scale bars = 250μm. Graphs showing osteoclast resorption surfaces (OC resorption surface) as a percentage of total cortical bone surface, osteoclast surface as a percentage of total bone perimeter (Oc.S/B.Pm) and the number of osteoclasts relative to bone perimeter (Oc.N/B.Pm) (mean ± SEM, n=3-4, per sex, per genotype).

**B.** Confocal images of proximal humerus double-labeled with calcein from P112 WT, *Slc20a2^+/-^* and *Slc20a2^-/-^* mice, scale bars = 1mm. Higher power images of double-labeled trabecular bone, scale bars = 10μm. Graphs showing trabecular mineralizing surface (MS), mineral apposition rate (MAR) and bone formation rate (BFR), (mean ± SEM, n=4 per sex, per genotype).

**C.** Images of double-labeled cortical bone, scale bars = 10μm. Graphs showing cortical mineralizing surface as a fraction of total bone perimeter (MS/B.Pm), mineral apposition rate (MAR) and bone formation rate (BFR) (mean ± SEM, n=4 per sex, per genotype).

**D.** Undecalcified proximal humerus sections from P112 WT, *Slc20a2^+/-^* and *Slc20a2^-/-^* mice stained with von Kossa/Paragon showing osteoid in pink, scale bars = 25μm. Graphs showing osteoid surface as a fraction of total bone perimeter (OS/B.Pm) and osteoid thickness (O.Th) (mean ± SEM, n=3-4, per sex, per genotype).

**Supporting Fig. S10: mRNA expression in primary chondrocyte and osteoblast cultures**

**A.** RT-qPCR analysis of genes involve in differentiation (*Col10a1*, *Runx2* and *Mmp13*), mineralization (*Alpl* and *Phospho1*) and phosphate transport (*Xpr1*, *Slc20a1* and *Slc20a2*) in high-density chondrocyte pellets from P7 WT, *Slc20a2^+/-^* and *Slc20a2^-/-^* mice (mean ± SEM, 2 independent experiments, n=3-5 per genotype). *Slc20a2* mRNA expression was reduced by approximately 50% in primary chondrocytes from heterozygous *Slc20a2^+/-^* mice and was undetectable in primary chondrocytes from homozygous *Slc20a2^-/-^* mice.

**B.** RT-qPCR analysis of genes involve in differentiation (*Ocn*, *Dmp1* and *Spp1*), mineralization (*Alpl* and *Phospho1*) and phosphate transport (*Xpr1*, *Slc20a1* and *Slc20a2*) in colony-forming calvarial osteoblasts from P6 WT, *Slc20a2^+/-^* and *Slc20a2^-/-^* mice (mean ± SEM, 3 independent experiments, n=1-4 per genotype). *Slc20a2* mRNA expression was reduced by approximately 50% in primary osteoblasts from heterozygous *Slc20a2^+/-^* mice and undetectable in osteoblasts from homozygous *Slc20a2^-/-^* mice.
