# Supplementary figures and images for: Slc20a2, Encoding the Phosphate Transporter PiT2, Is an Important Genetic Determinant of Bone Quality and Strength
Source: J Bone Miner Res. 2019 Mar 19;34(6):1101–14. doi: 10.1002/jbmr.3691 (PMC6618161; doi:10.1002/jbmr.3691)

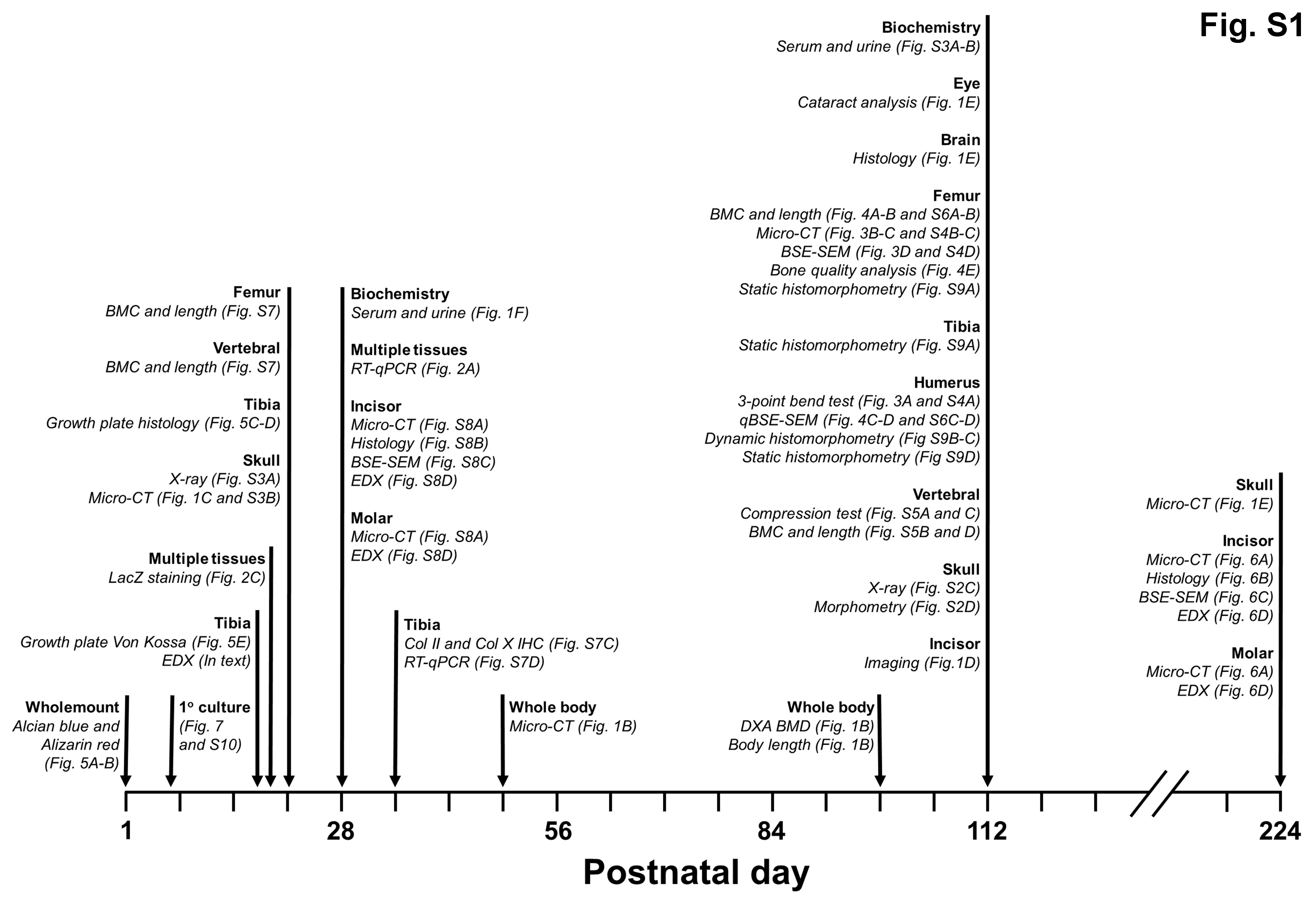

Supplement: Supplementary file 2 — Supporting Figure S1. [file JBMR-34-1101-s002.tif]

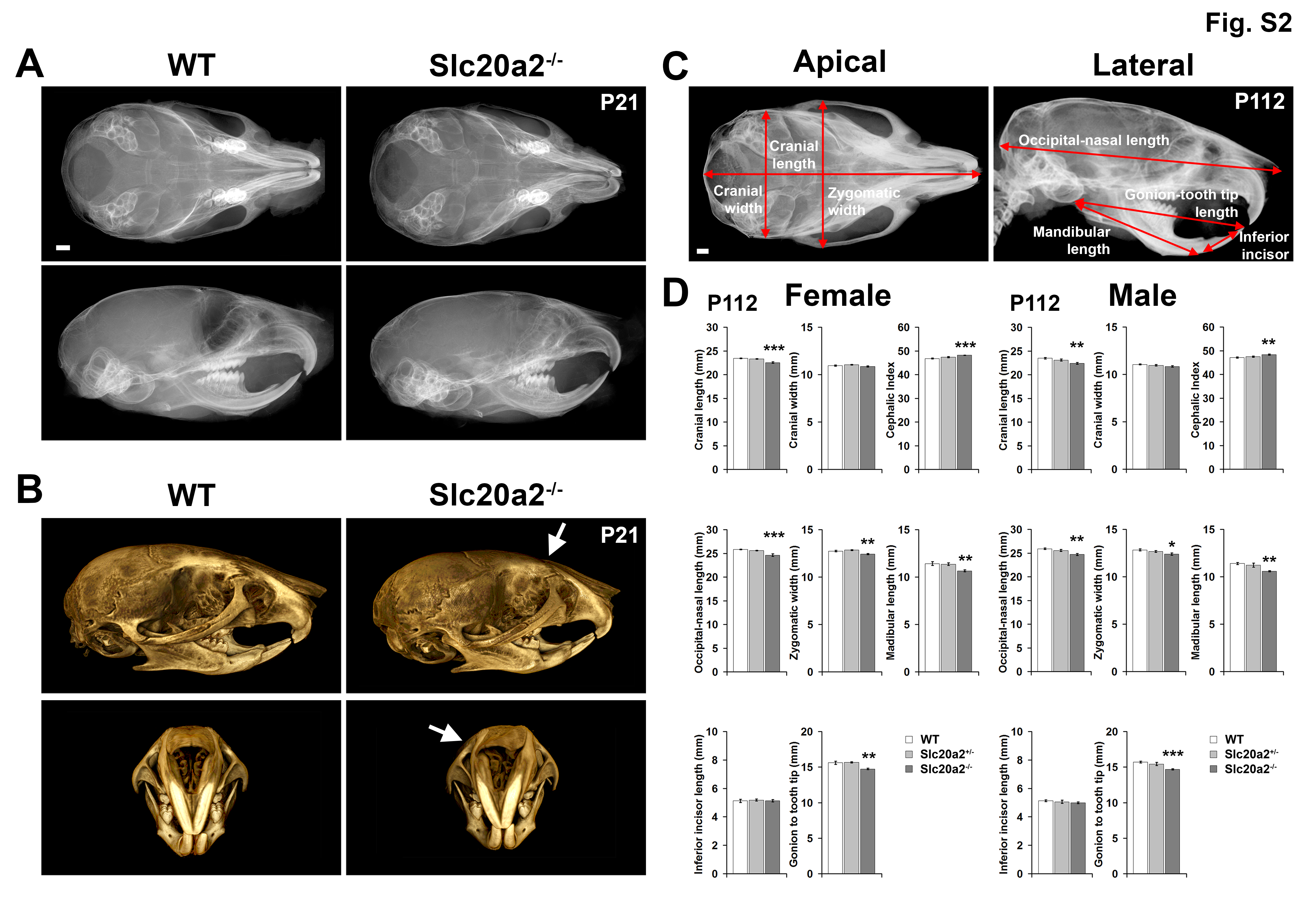

Supplement: Supplementary file 3 — Supporting Figure S2. [file JBMR-34-1101-s003.tif]

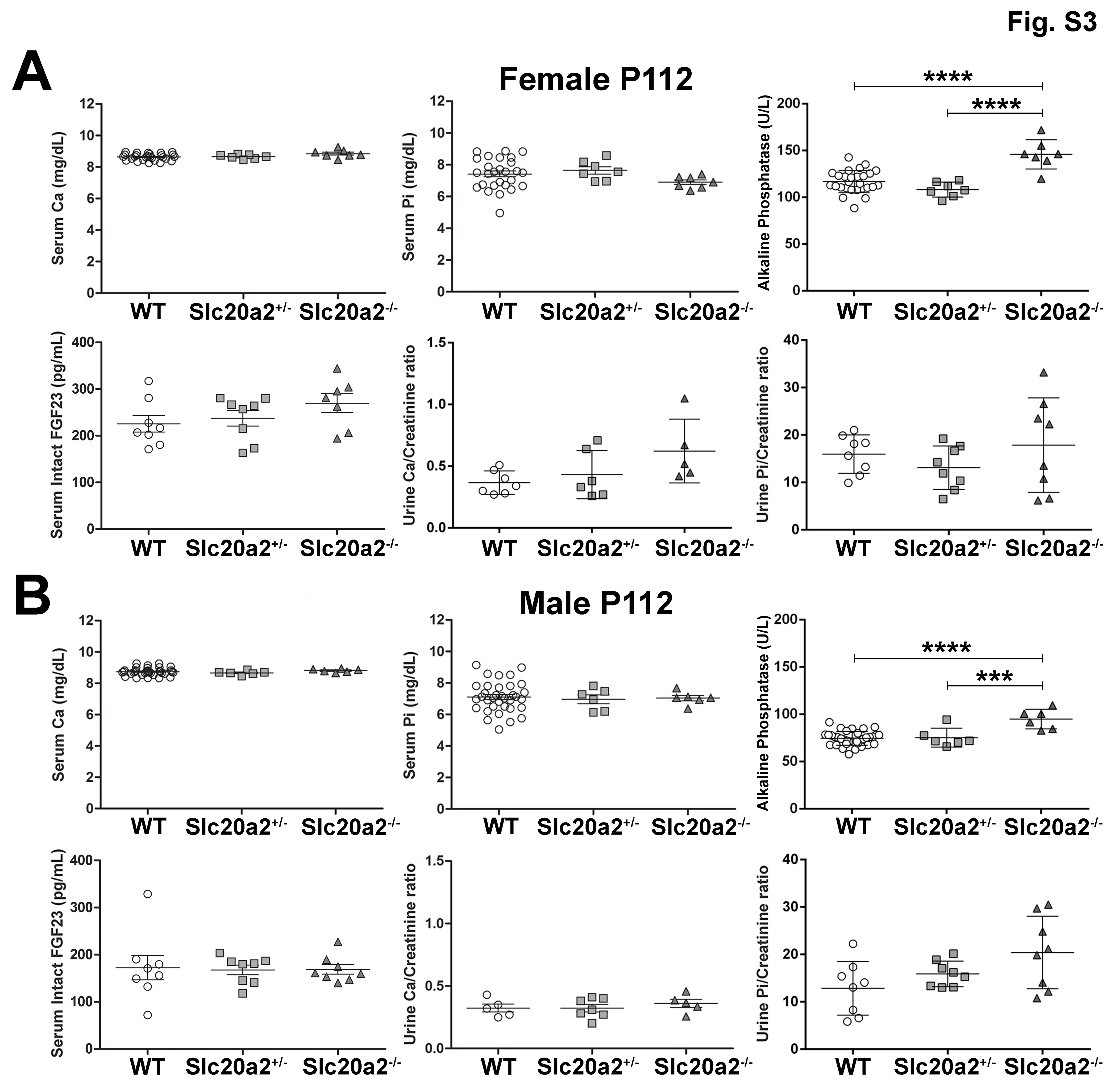

Supplement: Supplementary file 4 — Supporting Figure S3. [file JBMR-34-1101-s004.tif]

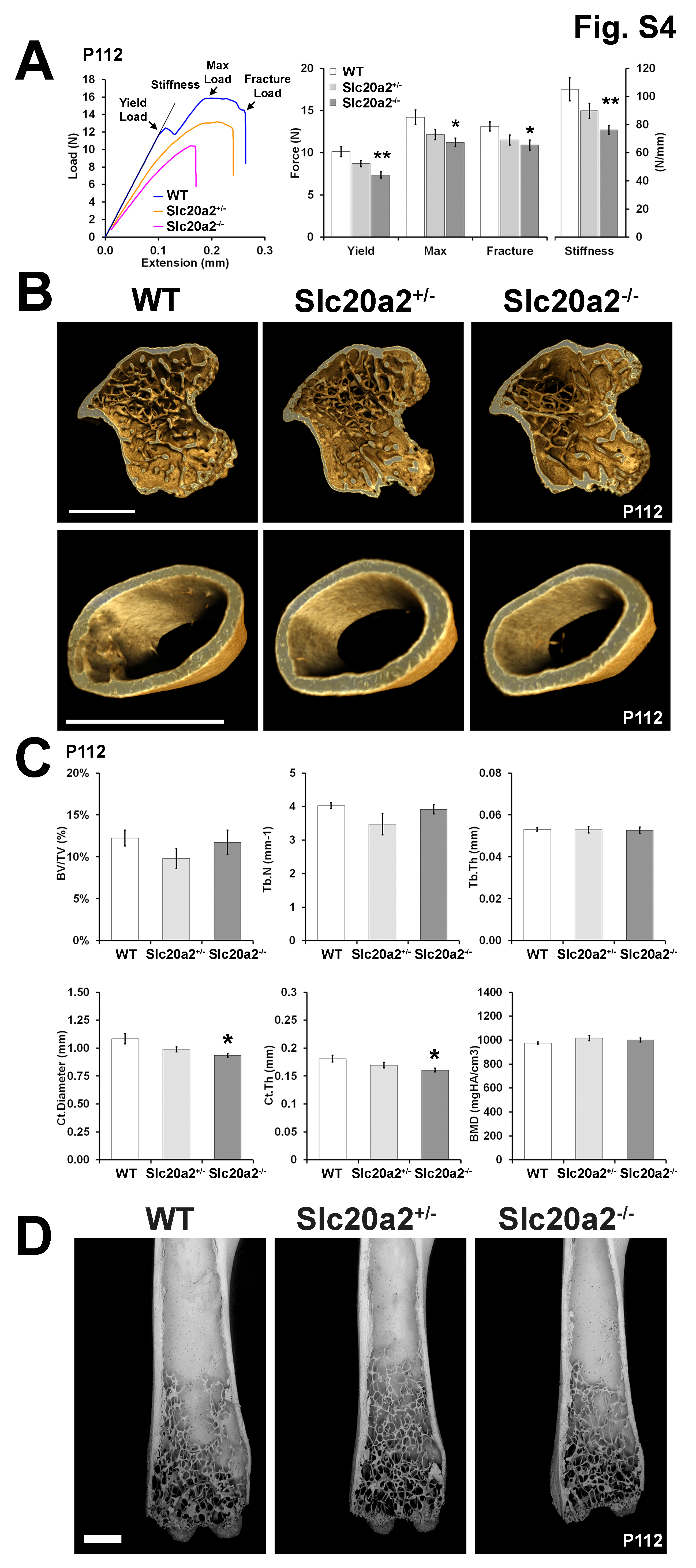

Supplement: Supplementary file 5 — Supporting Figure S4. [file JBMR-34-1101-s005.tif]

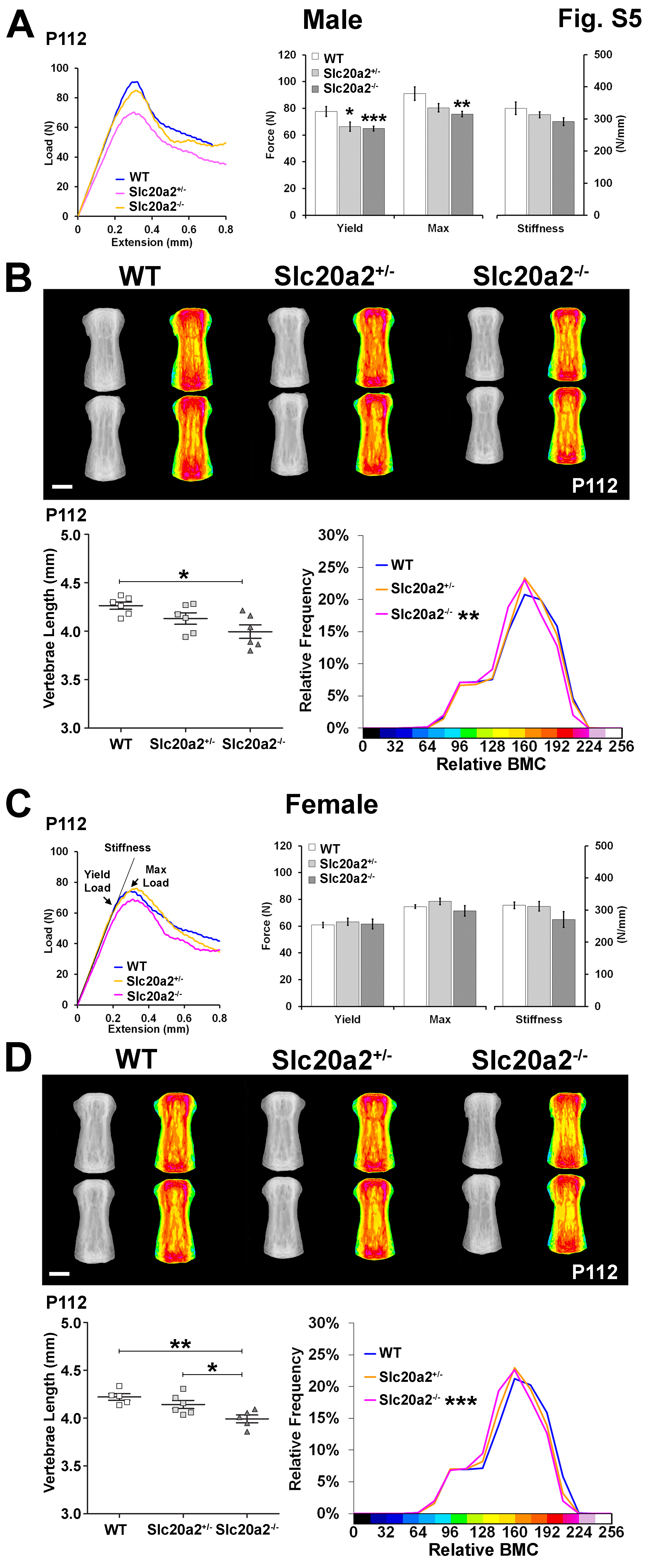

Supplement: Supplementary file 6 — Supporting Figure S5. [file JBMR-34-1101-s006.tif]

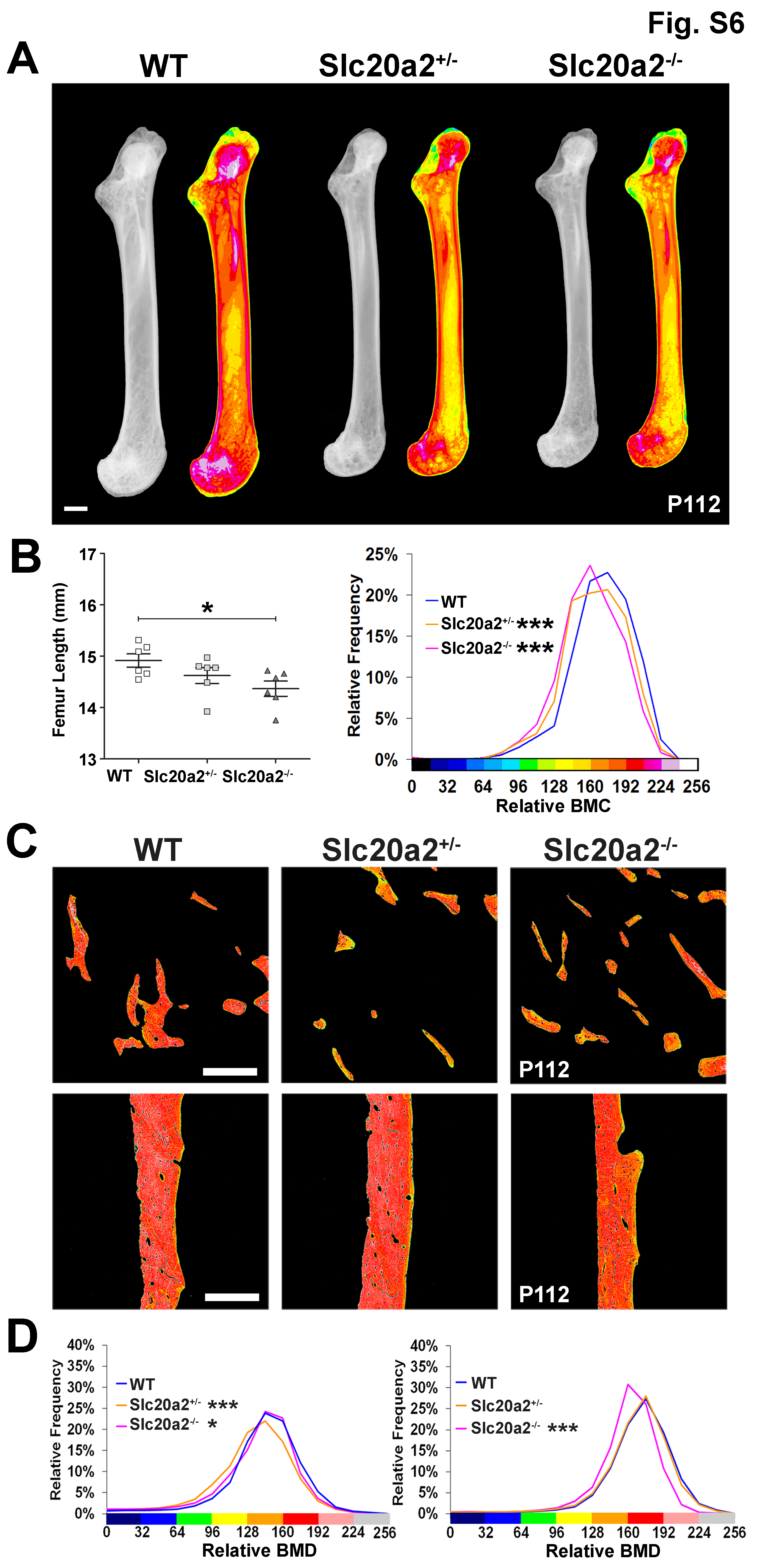

Supplement: Supplementary file 7 — Supporting Figure S6. [file JBMR-34-1101-s007.tif]

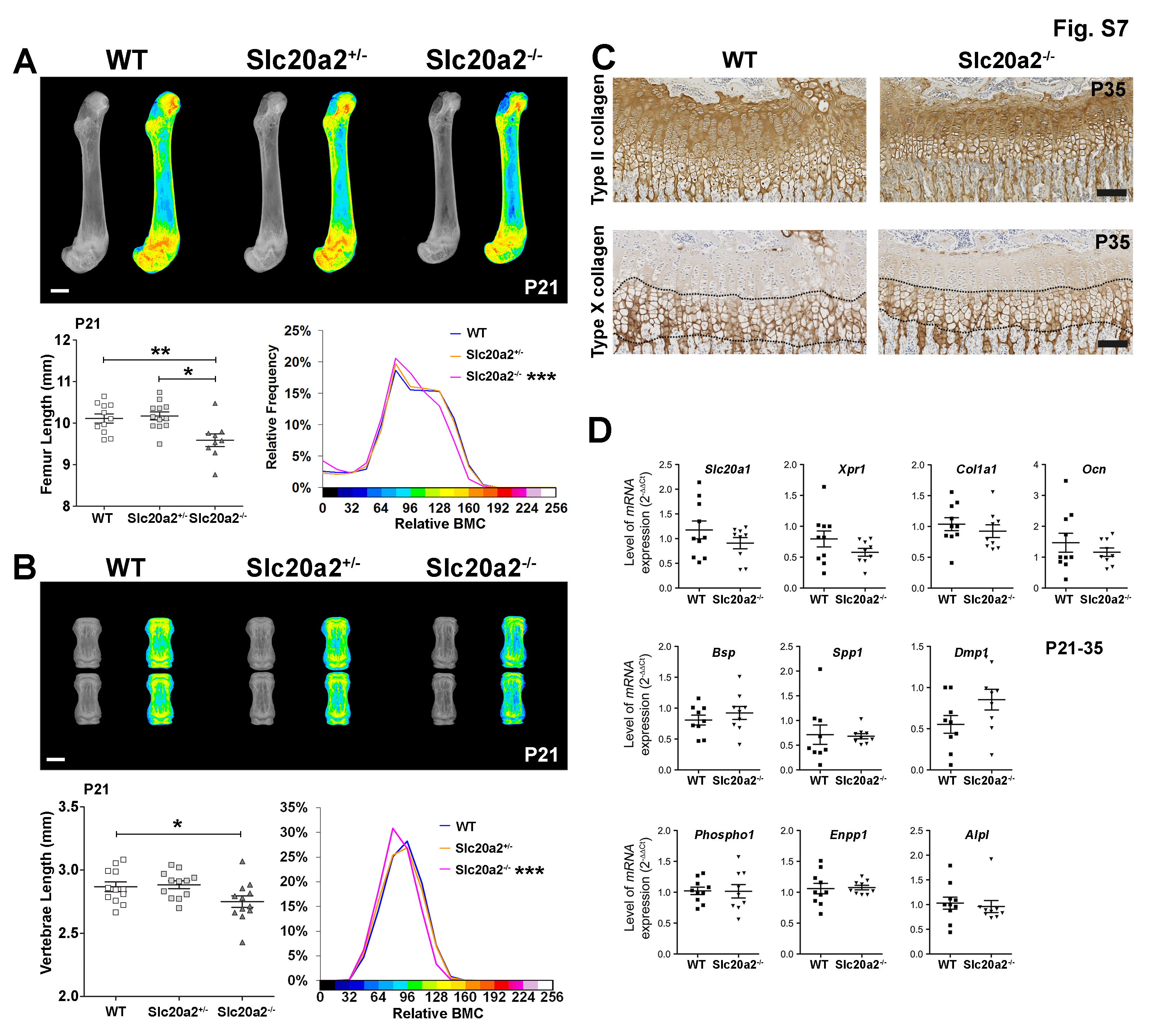

Supplement: Supplementary file 8 — Supporting Figure S7. [file JBMR-34-1101-s008.tif]

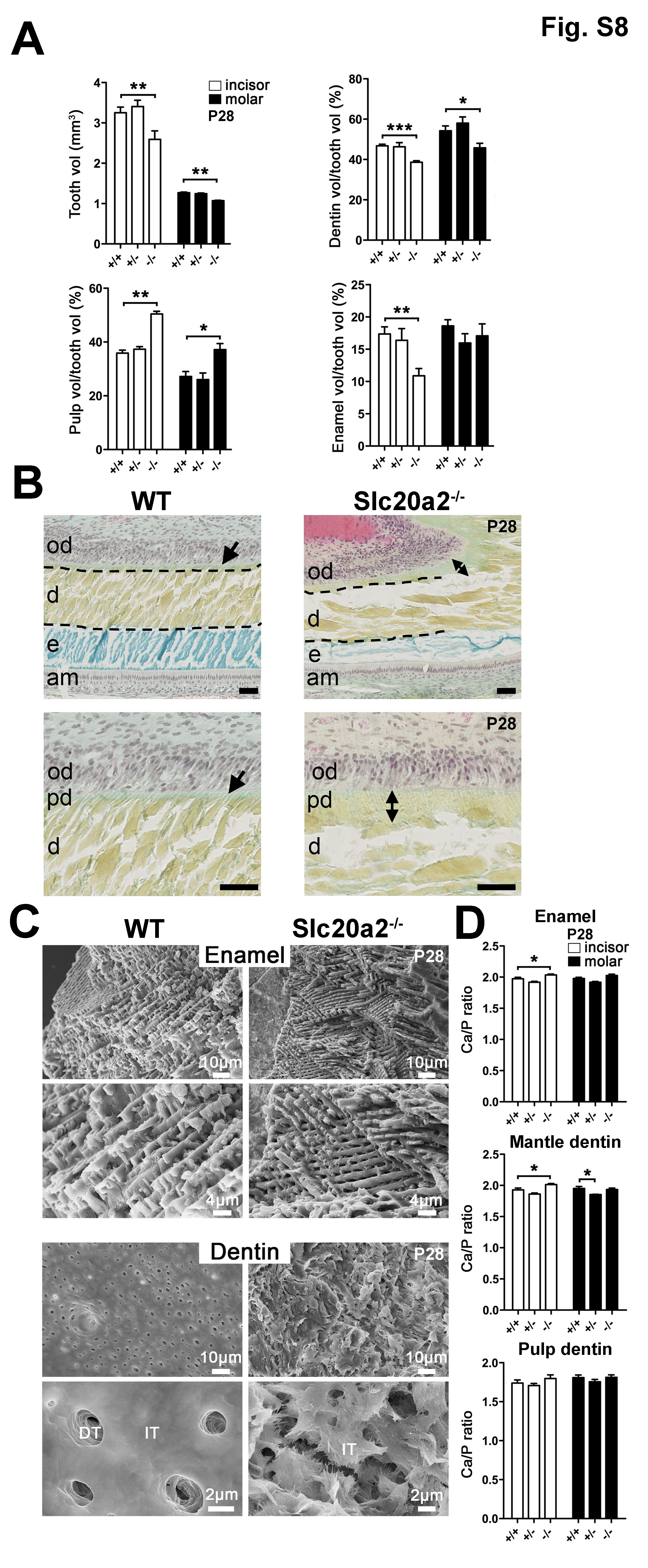

Supplement: Supplementary file 9 — Supporting Figure S8. [file JBMR-34-1101-s009.tif]

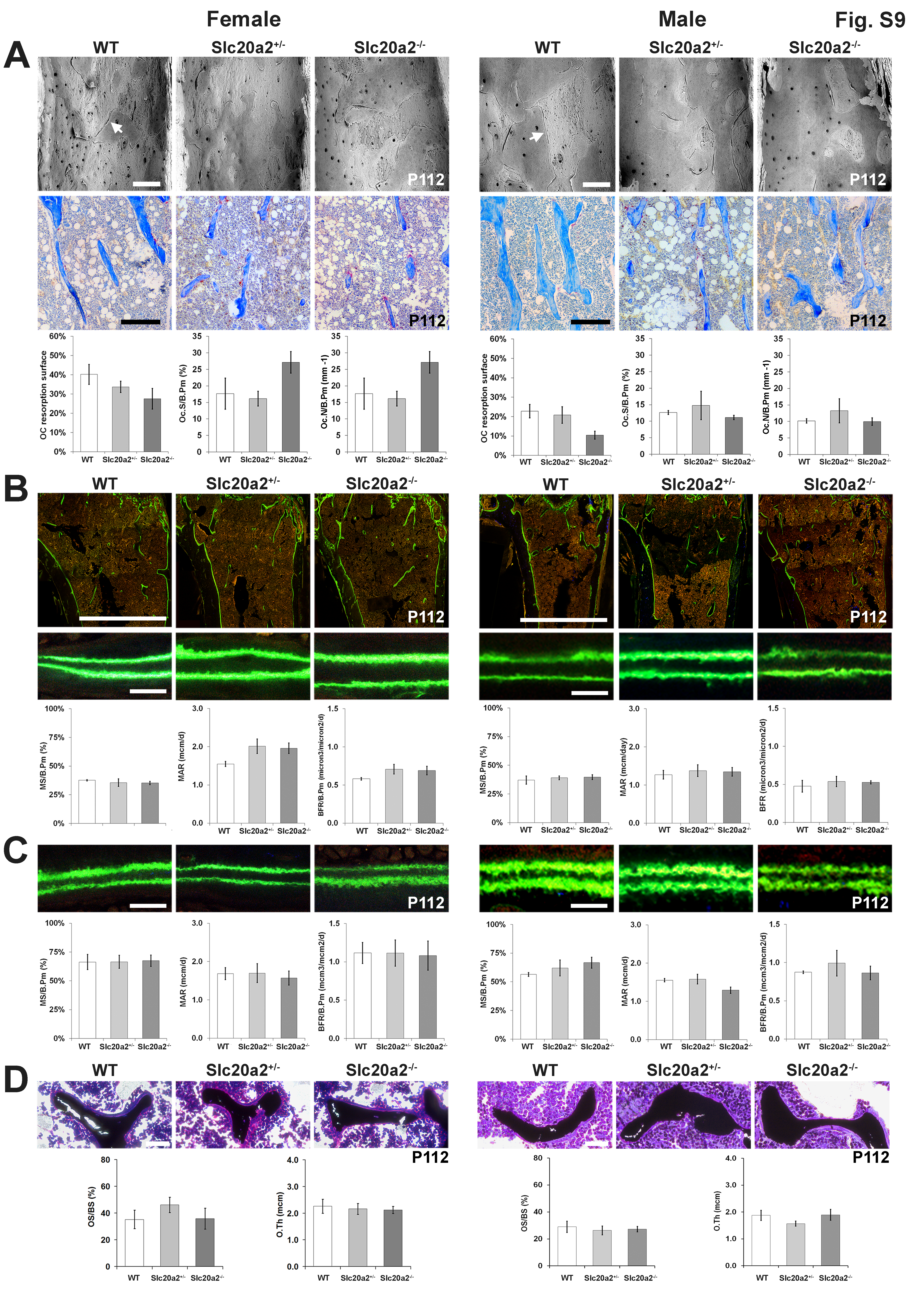

Supplement: Supplementary file 10 — Supporting Figure S9. [file JBMR-34-1101-s010.tif]

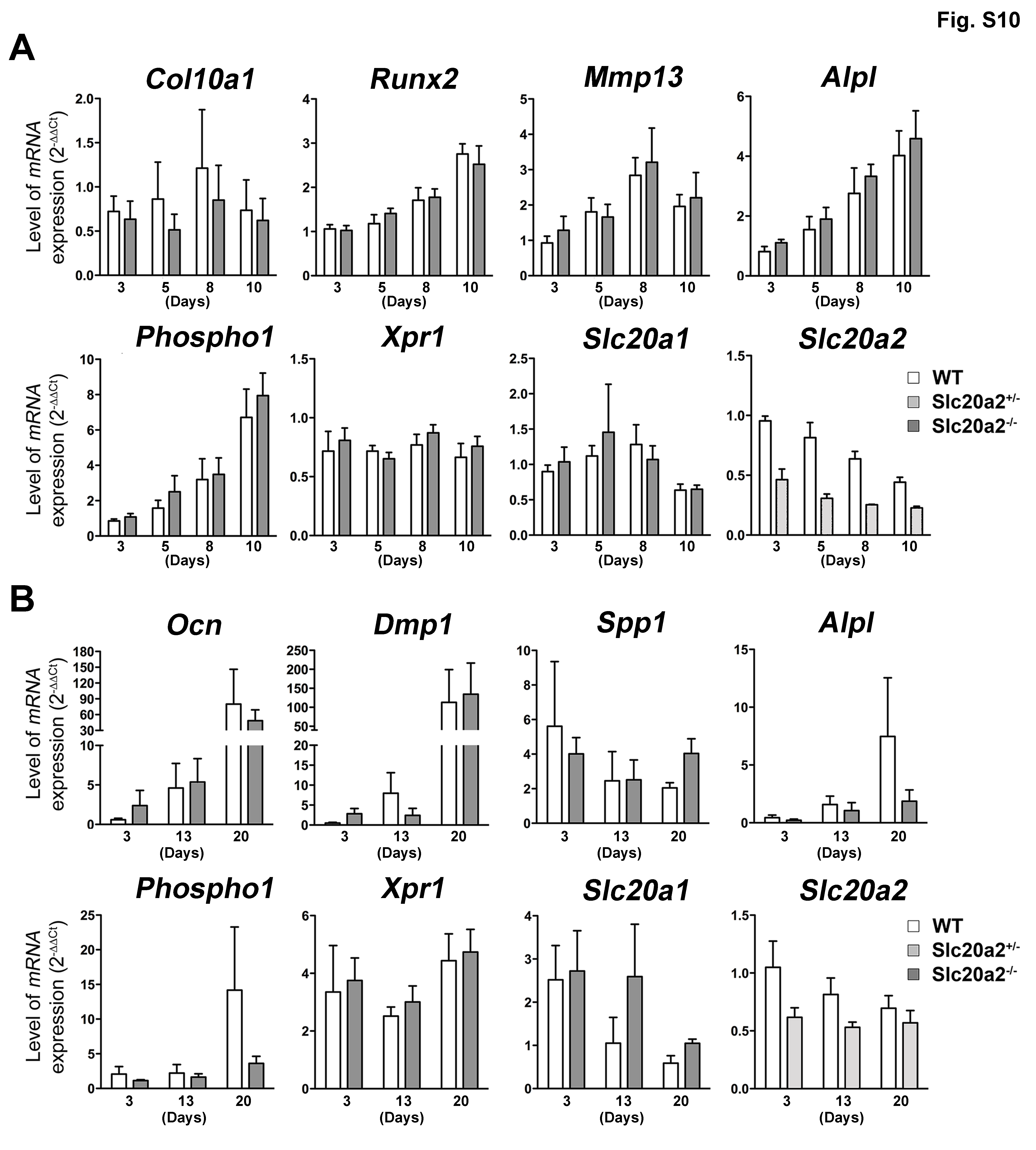

Supplement: Supplementary file 11 — Supporting Figure S10. [file JBMR-34-1101-s011.tif]
