# Supplementary material for: Slc20a2, Encoding the Phosphate Transporter PiT2, Is an Important Genetic Determinant of Bone Quality and Strength
Source: J Bone Miner Res. 2019 Mar 19;34(6):1101–14. doi: 10.1002/jbmr.3691 (PMC6618161; doi:10.1002/jbmr.3691)
Supplement: Supplementary file 12 — Supporting Table S1. [file JBMR-34-1101-s012.docx]

Supporting Table S1. List of antibodies

|  | | | **Immunohistochemistry** | |
| --- | --- | --- | --- | --- |
| Target protein | Supplier | Reference | Antigen retrieval | Primary antibody dilution |
| COL2A1 | MP Biomedical | 631711 | Proteinase K 20µg/mL 30 min 37°C | 1/200 |
| COL10A1 | Quartett | 2031501005 | Pepsin 0.1% in 0.5M acetic acid 2h at 37°C + Hyaluronidase 2 mg/mL 1h at 37°C | 1/100 |
