# Supplementary material for: Slc20a2, Encoding the Phosphate Transporter PiT2, Is an Important Genetic Determinant of Bone Quality and Strength
Source: J Bone Miner Res. 2019 Mar 19;34(6):1101–14. doi: 10.1002/jbmr.3691 (PMC6618161; doi:10.1002/jbmr.3691)
Supplement: Supplementary file 13 — Supporting Table S2. [file JBMR-34-1101-s013.docx]

**Supporting Table S2. List of primers.**

| **Genotyping PCR** | | | |
| --- | --- | --- | --- |
|  | | Forward (5’-3’) | Reverse (3’-5’) |
| *Slc20a2 WT allele* | | GGAGGAATGAGAGCAGCAAA | GGTCACCACAGCATGAGAAC |
| *Slc20a2 Null allele* | | GGAGGAATGAGAGCAGCAAA | CACAACGGGTTCTTCTGTTAGTCC |
| **Sybergreen RT-qPCR** | | | |
| Gene | | Forward (5’-3’) | Reverse (3’-5’) |
| *Alpl* | | GGCCAGCTACACCACAACA | CTGAGCGTTGGTGTTATATGTCTT |
| *Bsp* | | GAAAATGGAGACGGCGATAG | CATTGTTTTCCTCTTCGTTTGA |
| *Col1a1* | | GTCCTCCTGGCCCTGCTGGT | TCACCACGGTCTCCGGGAGC |
| *Col10a1* | | TGCTAGCCCCAAGACACAATAC | TGCCTTGTTCTCCTCTTACTGG |
| *Dmp1* | | ACCACCACCCACGAACAGTGAGT | AGAGTCCACCAGCCGGTCTGT |
| *Mmp13* | | CTATCCCTTGATGCCATTACCAG | ATCCACATGGTTGGGAAGTTC |
| *Ocn* | | TCTCTCTGACCTCACAGATGCCAAGC | GGACTGAGGCTCCAAGGTAGCG |
| *Runx2* | | GGACGAGGCAAGAGTTTCACC | GCTTCTGTCTGTGCCTTCTTGG |
| *Slc20a1* | | TGTGGCAAATGGGCAGAAG | AGAAAGCAGCGGAGAGACGA |
| *Slc20a2* | | CCATCGGCTTCTCACTCGT | AAACCAGGAGGCGACAATCT |
| *Spp1* | | CCCGGTGAAAGTGACTGATT | TTCTTCAGAGGACACAGCATTC |
| **Taqman RT-qPCR** | | | |
| Gene | Reference (Life Technologies) | | |
| *Enpp1* | Mm00501097_m1 | | |
| *GusB* | Mm01197698_m1 | | |
| *Phospho1* | Mm00462190_m1 | | |
| *Pnn* | Mm00447098_m1 | | |
| *Xpr1* | Mm00495501_m1 | | |
