# Supplementary material for: Slc20a2, Encoding the Phosphate Transporter PiT2, Is an Important Genetic Determinant of Bone Quality and Strength
Source: J Bone Miner Res. 2019 Mar 19;34(6):1101–14. doi: 10.1002/jbmr.3691 (PMC6618161; doi:10.1002/jbmr.3691)
Supplement: Supplementary file 14 — Supporting Table S3. [file JBMR-34-1101-s014.docx]

**Supporting Table S3. Tibial bone parameters in P21 *Slc20a2^-/-^* male mice.**

|  | ***Slc20a2^+/+^*** | ***Slc20a2^+/-^*** | ***Slc20a2^-/-^*** |
| --- | --- | --- | --- |
| **BV/TV (%)** | 7.372+/-0.086 | 7.368+/-0.251 | 6.678+/-0.722 |
| **Tb.N (mm-1)** | 1.948+/-0.030 | 2.062+/-0.050 | 1.967+/-0.211 |
| **Tb.Th (mm)** | 0.0379+/-0.0010 | 0.0357+/-0.0007 | **0.0339+/-0.0003 **** |
| **Ct.Th (mm)** | 135.7+/-1.8 | 128.3+/-9.8 | **107.2+/-4.5 *** |

**P*<0.05, ***P*<0.01 versus WT, ANOVA followed by Tukey’s post hoc test; n=3 WT, n=3 *Slc20a2^+/-^* and n=4 *Slc20a2^-/-^*.
